# Supplementary material for: Prospective longitudinal study of tobacco company adaptation to standardised packaging in the UK: identifying circumventions and closing loopholes
Source: BMJ Open. 2019 Sep 24;9(9):e028506. doi: 10.1136/bmjopen-2018-028506 (PMC6773294; doi:10.1136/bmjopen-2018-028506)
Supplement: Supplementary data [file bmjopen-2018-028506supp001.pdf]

Supplementary file material for “Assessing tobacco company adaptation to standardised packaging: identifying circumventions and closing loopholes”

Table S1. Tobacco advert characteristics by price segment (n= 195) [1]

	FM & RYO				Cigars/ cigarillos	Pipe
Characteristics of advert[2]	Subvalue[3]	Value	Midprice	Premium		
Type of tobacco						
FM	22	78	42	21		
RYO	n/a	24	13	16		
Characteristics promoted						
New brand variant[4]	7	23	5	1	0	0
New packaging	4	25	17	16	0	0
Limited Edition	0	4	5	5	2	0
Filter	0	16	0	6	0	0
Price	19	68	14	4	2	1
Retailer profit	1	42	21	5	6	1

[1] Totals do not add up to 195 adverts as many adverts promoted more than one type of tobacco, more than one price segment and more than one characteristic in the same advert.

[2] Does not include cigars/cigarillos or pipe tobacco or as we have no data on price segment for these products.

[3] FM only. This price segment does not exist for RYO.

[4] There were 38 adverts in total for a new brand variant but 2 of the adverts could not be categorised by price segment.

Evans-Reeves KA, *et al. BMJ Open* 2019; 9:e028506. doi: 10.1136/bmjopen-2018-028506

Table S2 Industry strategies to circumvent standardised packaging and exploit loopholes implied by commercial literature

Industry strategy	Tactic	Quote from the commercial literature
1. Keep branded packaging on the market throughout the sell-off period	1.1 Over-producing branded packs before the sell-off	"BAT has had to produce higher stocks prior to the 20 May 2016 manufacturing deadline in order to avoid out of stocks [branded]…The TPD explicitly provides for transitional provisions to allow product manufactured before 20 May 2016 to be sold until 20 May 2017." Ron Ridderbeek, head of legal and external affairs at British American Tobacco [*Retail Newsagent*, 10 May 2016]
	1.2 Introducing smaller pack sizes just before the sell-off	"Tinkering with pack sizes is a proven wheeze for off setting the impact of tobacco's growing tax burden on price per pack. JTI has managed to hit the £6.50 price point by launching 17-packs of Sovereign Blue, Sky Blue, Green and Dual. And it's just in time; from May, pack sizes will be limited to a minimum of 20 cigs." Japan Tobacco International [*The Grocer,* 11 February 2016]<br><br>"Launching next month as the European Union Tobacco Productive Directive comes into force, [Pall Mall's] redesign - available in 10-pack and 18-pack King Size and Super King 18s - will include the new Taste Plus Filter … price-marked packs are set to be available at the current [price]." [*The Grocer,* 18 April 2016]
	1.3 Providing financial incentives to retailers to sell a particular company's brands	"Retailers have reported a "massive" increase in cigarette sales as tobacco manufacturers cut prices and offer cash incentives to bolster brands at a time when new plain packaging and smaller pack regulations are set to shake up the industry. … An independent retailer… said: "I have seen a massive increase in sales. We were also given PoS including shelf wobblers to encourage as many customers as possible to buy the brand. I think this is a really smart way of driving sales and is something that retailers will respond to." Meanwhile, Imperial Tobacco will knock 10p off the price of four of its biggest tobacco brands - Lambert & Butler, JPS, Players and Gold Leaf - from 1 June, while maintaining cash margins... [A retailer who] has been trialling the new Imperial prices at his store… said: "We sold up to one and a half extra outers a week because of the lower price." [*Retail Newsagent,* 26 May 2016]<br><br>"A value brands price war on cigarettes is hurting retailers' margins and could lead to stores being "lumbered" with a heap of unwanted, unsaleable stock [with legislation changes;] tobacco suppliers have been dropping the prices of cigarette packs at the value end of the market… some manufacturers… selling value cigarettes at a loss to gain market share… Last runs of branded packs are price-marked lowly and [a retailer] told Retail Express that the actions of suppliers will have grave consequences for unsuspecting retailers.<br><br>"Situations will arise where retailers are taken in by the low, price-marked RRPs and buy in bulk, thinking that consumers will jump at the chance to catch the last batches of cheap packs…Come May 20, they'll find themselves lumbered with packs of cigarettes they can no longer sell, as supply will outstrip demand…It's a

		unique situation, but the suppliers have stock they need to get rid of. They're cutting the prices down and loss-leading on them [but]Independent retailers certainly can't afford to loss-lead. "…[Another retailer] said: "… Companies downtrading to push their own agendas does not help us at all. I've even heard that certain wholesalers were offered better percentages to buy in these packs, which would obviously be recouped at the other end.""[*Better Retailing,* 17 February 2017]<br><br>""The tobacco suppliers will themselves take back any branded packs after the May cut-off," [wholesaler manager] told RN. "They have teams of people calling on retailers and they are notifying them as to the process. They are saying if they have any branded stock left to call their local rep and they will come in and replace it." [*Retail Newsagent*, 2 March 2017]
2. Maintain brand differentiation through systematic name changes		"We recognise that consumers use brand and colour when asking for their pack of cigarettes." Therefore, generally, those products traditionally presented as 'full flavour' would have 'real' or 'original' added to their names and be denoted predominantly by the colour 'red'. All brand variants with the flavour descriptor 'smooth' would change to 'bright' and have 'blue' added, 'menthol' would become 'green' and 'capsule' would become 'crushball'." [*Retail Newsagent*, 28 October 2016]
3. Focusing on lower price segments	Introducing premium features into cheaper price segments	"Living in a post-EUTPD II world… [a retailer reported] "Customers have become more price-sensitive since legislation banned the sale of 10- packs and they are more concerned about how much they can get for their money these days"…To help retailers tap into the trend for budget tobacco, JTI has relaunched Scottish brand Kensitas Club King Size and dropped its RRP " Yau A. The latest developments from the biggest tobacco brands. [*Retail Newsagent*, 5 April 2018]
4. Promote products by innovating exemptions to the legislation	4.1 Bevelled edges and resealable packs	"In order to re-engage disengaged cash-strapped smokers migrating to lower-priced brands and categories, manufacturers have been "adding value" to their brands through innovation, both on the pack and via the product itself… PMI concentrated on areas such as pack size (extra-large pack sizes), pack type (resealable), cigarette length (compact), microslims, filter type (with a focus on recessed filters) and flavour capsules (including the world's first recessed filter capsule and the world's first double capsule – menthol and peppermint). Pack innovation: Tactile finishes, including raised surfaces such as embossing and bevelled edges… (39)[1] [*Euromonitor*, July 2016]
	4.2 Menthol	"JPS Green Edge will replace it and feature a menthol inner liner within the packaging, which the company claims will be "more effective at transferring flavour to the cigarettes than traditional menthol tips"". Imperial Tobacco [*Talking Retail*, 24 November 2017]

[1] This Euromonitor report was published two months into the sell-off period. We did find evidence of embossing and raised surfaces on packets prior to the full implementation of the legislation but these changes were lost when standardised packaging came into effect. During the sell-off, filter innovations, capsule innovations, bevelled edges and a pack resealing mechanism on one brand were observed – these changes were able to carry through when packs became standardised. In this study we did not observe extra length cigarettes, a greater volume of cigarettes or microslims.

Evans-Reeves KA, *et al. BMJ Open* 2019; 9:e028506. doi: 10.1136/bmjopen-2018-028506

	4.3 Filters (another way to create the perception that some products are healthier than others?)	Marlboro Touch, previously known as Marlboro Gold Touch, now has a soft touch pack and firm filter keeping the consistency with the rest of the Marlboro range. The firm filter allows adult smokers a cleaner way to stub out their cigarette, as well as adding an overall feeling of quality, without having an impact upon taste." [*Talking Retail,* 31 July 2015]<br><br>"Launching …as the European Union Tobacco Productive Directive comes into force, [Pall Mall's] redesign… will include the new Taste Plus Filter that aims to create a firmer in-hand feel and improve filtration" [*The Grocer,* 18 April 2016]<br><br>"…JPS Triple Flow… "a revolution in cigarette technology." Triple Flow uses "unique" "easy draw" channels, with a new, "high quality smooth" tobacco blend enhanced by a mineral filter that provides "a fuller smoking experience". Each cigarette is also wrapped in a special paper, designed to actively reduce the amount of smoke emitted - which may result in less smell, says Imperial. Brand portfolio manager Emma Stew said: "Smooth blends now account for almost a quarter [ITUK estimates September 2015] of factory made cigarette (FMC) sales, and Triple Flow has been designed to offer the ultimate smooth smoking experience. 'With its next generation features and distinctive packaging, there is nothing like it in the market'" [*Wholesale News* November 2015]
	4.4 RYO papers and filter tips	Imperial Tobacco brand manager Jamie Burns-Smith said: "Natura taps into an increasing consumer desire for responsibly sourced products. Crafted from 100% hemp, Natura papers and tips are the perfect sustainable way to enjoy an authentic tobacco taste. Imperial Tobacco has also launched Rizla Menthol Tips, as the company claims "menthol tips are the fastest-growing segment of the tips market". [*Talking Retail,* 3 August 2017]
	4.5 Cigars	"Break, as they are cigarillos, are exempt. We can do packs of less than 20, and we can do flavours… Cigars won't be a standard colour pack, and will still have product name branding… you can sell individually and in 10 packs" Jens Christiansen, Scandinavian Tobacco Group's head of marketing & public affairs [*Retail Express*, 6 June 2016]<br><br>"Cigars benefit from the fact they can be sold in 10-packs, while also being exempt from certain branding restrictions. Scandinavian Tobacco Group (STG) is using this to its advantage and its marketing this year… the packaging stands out when compared to traditional cigarettes… and the investment made by companies such as STG to raise awareness and education in the convenience trade really helps... Ritmeester's says miniature cigars have traditionally been products that are better known among tobacco enthusiasts. As a result, it is offering samples of its Royal Dutch range to retailers to increase awareness…

Evans-Reeves KA, *et al. BMJ Open* 2019; 9:e028506. doi: 10.1136/bmjopen-2018-028506

		"I find the curry socials Ritmeester holds with retailers and the trade press very useful. It's a hands-on, but informal way of learning about the latest developments in the cigar market. There isn't as much awareness about cigars as cigarettes. These opportunities give us a chance to learn about how we can upsell to smokers who want value." [Retailer]" [*Retail Newsagent*, 5 April 2018]<br><br>"If a smoker only has £5 on them, they cannot buy from your gantry, but if you present them with cigars they can." Ritmeester [*Retail Express*, 17 September 2017]<br><br>"[Retailer] says he struggles with selling Mini Moods. [Ritmeester's] Lyles explains: "It might be because you're aiming them at cigar smokers, but it's primarily aimed at cigarette smokers. Because of the double filter, it makes for a smoother smoke." … Cigars are now the cheapest product in a gantry. Lyles says: "If a smoker only has £5 on them, they cannot buy from your gantry, but if you present them with cigars they can." [*Retail Express*, 17 September 2017]<br><br>"'The margins we make on miniature cigars are 30% when sold at RRP' [Retailer]" [*Retail Newsagent*, 5 April 2018]
	4.6 Pipe tobacco sold as RYO	"The sell-through period for branded RYO and cigarettes ended last week, with products now sold in minimum formats and carrying larger health warnings as part of the new tobacco legislation. Yet, UK tobacco manufacturer Gawith Hoggarth is promoting its Kendal brand as a product which is cut to pipe tobacco regulations but can also be used as hand-rolling tobacco. "We've cut the tobacco to the thinnest width we are allowed to which means we can sell it in branded packaging and in 12.5g pouches," said the company's area sales manager Martin Bobbette. The company hopes retailers will recommend the product to smokers who ask for "whatever is cheapest"" [*Retail Newsagent*, 25 May 2017]

Evans-Reeves KA, *et al. BMJ Open* 2019; 9:e028506. doi: 10.1136/bmjopen-2018-028506

Table S3 Compliance with standardised packaging over time[1]

	% standardised packs	change from previous month	total sticks sold	non-compliant sticks sold
Jul-16	0%		2,574,340,308	2,574,204,015
Aug-16	0%	0%	2,620,104,622	2,610,727,340
Sep-16	1%	1%	2,424,692,602	2,397,886,927
Oct-16	3%	2%	2,491,577,487	2,423,864,794
Nov-16	4%	1%	2,424,660,803	2,323,057,266
Dec-16	9%	4%	2,593,371,625	2,370,713,888
Jan-17	21%	12%	2,316,632,301	1,829,871,306
Feb-17	45%	24%	2,184,811,712	1,204,205,686
Mar-17	64%	20%	2,402,514,817	852,983,422
Apr-17	79%	14%	2,386,041,031	506,014,392
May-17	91%	12%	2,475,338,965	228,361,777
Jun-17	97%	6%	2,359,196,539	81,803,934
Jul-17	98%	1%	2,461,000,857	53,453,290
Aug-17	98%	0%	2,412,267,420	43,336,437
Sep-17	99%	0%	2,267,864,526	29,804,540
Oct-17	99%	0%	2,303,639,526	27,803,694
Nov-17	99%	0%	2,231,013,199	25,718,138
RYO			total thousands of kg sold	non-compliant* (thousands of kg sold)
Aug-16	1%		552287	546963
Sep-16	1%	0%	521917	516721
Oct-16	11%	10%	528560	469493
Nov-16	18%	7%	518188	424040
Dec-16	26%	8%	541359	400109
Jan-17	44%	18%	516174	288710
Feb-17	55%	11%	490464	218940
Mar-17	75%	20%	539695	132601
Apr-17	91%	16%	534343	45509
May-17	97%	5%	557734	19294
Jun-17	98%	2%	539118	9037
Jul-17	99%	1%	559731	5908
Aug-17	99%	0%	560994	4609
Sep-17	99%	0%	536870	2984
Oct-17	99%	0%	542052	2802
Nov-17	100%	0%	530219	2511

*RYO estimated to be sold in standardised packaging if sold in 30g or larger not pricemarked pack and (a) 30g pack of same variant on the market or (b) if 30g pack never available, pack on the market at end of analysis period)

Evans-Reeves KA, *et al. BMJ Open* 2019; 9:e028506. doi: 10.1136/bmjopen-2018-028506

Table S4 Menthol and capsule innovation

	Present at start		New Variant		Total	
	N	%	N	%	N	%
Menthol*						
FM premium	9	28%	2	20%	11	26.2
FM midprice	8	25%	0	0%	8	19.0
FM value	8	25%	4	40%	12	28.6
FM subvalue	3	9%	3	30%	6	14.3
no price segment	4	13%	1	10%	5	11.9
	32	76.2%	10	23.8%	42	100.0
Capsule**						
FM premium	2	15%	0	0%	2	10.5
FM midprice	2	15%	0	0%	2	10.5
FM value	9	69%	3	50%	12	63.2
FM subvalue	0	0%	3	50%	3	15.8
Total	13	68.4%	6	31.6%	19	100.0

*Menthol: variant name includes 'menthol' or 'green'

**Capsule: variant name includes 'capsule', 'crushball', 'dual', 'duo', 'demand', 'click on' or 'burst'

Table S5 Euromonitor estimates of the growth of cigars and cigariillos

	value constant £ mln	value current £ mln	volume million units	
2015	291.8	289.9	390.4	
2016	282.5	282.5	382.8	
2017	324.5	332.6	451.6	estimate
2018	347.8	366.3	493.8	estimate

Source: © Euromonitor International

Evans-Reeves KA, *et al. BMJ Open* 2019; 9:e028506. doi: 10.1136/bmjopen-2018-028506
