# Supplementary material for: Prospective longitudinal study of tobacco company adaptation to standardised packaging in the UK: identifying circumventions and closing loopholes
Source: BMJ Open. 2019 Sep 24;9(9):e028506. doi: 10.1136/bmjopen-2018-028506 (PMC6773294; doi:10.1136/bmjopen-2018-028506)
Supplement: Author's manuscript [file bmjopen-2018-028506.draft_revisions.pdf]

BMJ Open is committed to open peer review. As part of this commitment we make the peer review history of every article we publish publicly available.

When an article is published we post the peer reviewers' comments and the authors' responses online. We also post the versions of the paper that were used during peer review. These are the versions that the peer review comments apply to.

The versions of the paper that follow are the versions that were submitted during the peer review process. They are not the versions of record or the final published versions. They should not be cited or distributed as the published version of this manuscript.

BMJ Open is an open access journal and the full, final, typeset and author-corrected version of record of the manuscript is available on our site with no access controls, subscription charges or pay-per-view fees (http://bmjopen.bmj.com).

If you have any questions on BMJ Open's open peer review process please email info.bmjopen@bmj.com

BMJ Open

Assessing tobacco company adaptation to standardised packaging: identifying circumventions and closing loopholes

Journal:	*BMJ Open*
Manuscript ID	bmjopen-2018-028506
Article Type:	Research
Date Submitted by the Author:	17-Dec-2018
Complete List of Authors:	Evans-Reeves, Karen; University of Bath, Tobacco Control Research Group, UK Centre for Tobacco and Alcohol Studies, Department for Health<br>Hiscock, Rosemary; University of Bath, Tobacco Control Research Group, UK Centre for Tobacco and Alcohol Studies, Department for Health<br>Lauber, Kathrin; University of Bath, Tobacco Control Research Group, UK Centre for Tobacco and Alcohol Studies, Department for Health<br>Gilmore, Anna; Tobacco Control Research Group, UK Centre for Tobacco and Alcohol Studies, Department for Health, University of Bath
Keywords:	PUBLIC HEALTH, PREVENTIVE MEDICINE, TOBACCO, STANDARDISED PACKAGING
Note: The following files were submitted by the author for peer review, but cannot be converted to PDF. You must view these files (e.g. movies) online.	
Video 1.MP4	

SCHOLARONE™ Manuscripts

Assessing tobacco company adaptation to standardised packaging: identifying circumventions and closing loopholes

Karen A Evans-Reeves[1], email K.A.Evans-Reeves@bath.ac.uk

Rosemary Hiscock[1*], email R.Hiscock@bath.ac.uk

Kathrin Lauber[1], email K.Lauber@bath.ac.uk

Anna B Gilmore[1], email A.Gilmore@bath.ac.uk

[1]Tobacco Control Research Group, University of Bath and UK Centre of Tobacco and Alcohol Studies, Bath, UK

*Corresponding author: +44 (0)1225 388388

Key words: tobacco, tobacco industry, standardised packaging, plain packs

Word count: 4251

For peer review only

Abstract

Objectives: UK standardised packaging legislation was introduced alongside pack size and product descriptor restrictions of the European Union Tobacco Products Directive to end tobacco marketing and misinformation via the pack. This paper aims to assess compliance with the restrictions and identify attempts to continue to market tobacco products and perpetuate misperceptions of harm post-legislation.

Design, setting and intervention: A prospective study of the introduction of standardised packaging of tobacco products to the UK.

Participants and outcomes: We analysed Nielsen sales data to assess whether the legally required changes in pack branding, size and name were implemented by the deadline. To explore any adaptations to products and packaging we analysed data from monthly pack purchases of factory-made and roll-your-own cigarettes, tobacco adverts from retail trade magazines and articles on tobacco from commercial literature (e.g. trade, market analyst and tobacco company publications).

Results: While compliance was good (99% by November 2017), tobacco companies used the one-year sell-through to their advantage by communicating brand name changes and providing financial incentives for retailers to buy large volumes of branded packs. They made adaptations to tobacco products to enable continued marketing and brand differentiation. For example, companies included colours (either to convey the historical colour of the pack or a characteristic) arguably enabling continued misperceptions about the relative harms of products. In addition, innovations to exemptions of the legislation, namely filters, packaging edges, seals, multipack outers, RYO accessories, cigars and pipe tobacco were all marketed to retailers.

For peer review only - http://bmjopen.bmj.com/site/about/guidelines.xhtml

Conclusions: Tobacco companies adapted to packaging restrictions by innovating their marketing activities. These findings should enable policy makers globally to close loopholes.

For peer review only

For peer review only - http://bmjopen.bmj.com/site/about/guidelines.xhtml

Article Summary

Strengths and limitations of the study

- The strength of this paper lies both in the detail and depth of each analysis including our systematic analysis of retail press adverts (n=195) and commercial literature articles and reports (n= 396) alongside pack purchases of the top selling brands and detailed sales information from Nielsen.
- Using multiple data sources has, in many instances, enabled findings to be verified by more than one source and enabled a greater understanding of the tobacco industry's motives for any changes made to their products and packaging.
- Resources prevented us from acquiring Nielsen data on cigars or pipe tobacco and from purchasing more than one brand per price segment in the pack purchasing study element but we were able to compensate, to some extent, by using information available from other sources.
- We assumed that 30g RYO packs were always in standardised packs and that larger packs would switch to standardised packs at the same time; the temporal patterns of switching names and sizes are similar to FM (where standardised packaging was indicated) and it is therefore reasonable to assume that branding was removed at the same time.
- Although, we do not know the extent to which Nielsen variant name changes lag behind and even reflect those printed on packs in retailers, the main name change patterns found in the Nielsen data were similar to those found in a UK convenience store study and an evaluation of the introduction of standardised packaging in Australia.

For peer review only

For peer review only - http://bmjopen.bmj.com/site/about/guidelines.xhtml

Funding: This work was supported by Cancer Research UK grant number C27260/A23168. The authors are members of the UK Centre for Tobacco & Alcohol Studies, a UK Clinical Research Collaboration Public Health Research Centre of Excellence whose work is supported by funding from the Medical Research Council, British Heart Foundation, Cancer Research UK, Economic and Social Research Council, and the National Institute for Health Research under the auspices of the UK Clinical Research Collaboration (MR/K023195/1).

Competing Interests: The authors have no competing interests.

For peer review only

For peer review only - http://bmjopen.bmj.com/site/about/guidelines.xhtml

Background

Standardised tobacco packaging, one of the most significant policy threats to the tobacco industry in recent years, came into force in the UK on May 20th 2016. From this date all new factory made (FM) cigarettes and roll your own (RYO) tobacco, manufactured or imported for UK consumption were to be sold in standardised packaging. Tobacco companies were given until May 20th 2017 to comply with the law; a period referred to as the 'sell-through'. During this time, companies were not permitted to print any new branded packets(1, 2). The UK legislation(1) was implemented in concert with the revision of the 2001 European Union (EU) Tobacco Products Directive (TPD)(3) which placed further restrictions on packaging and naming of brands (Box 1)(3).

After Australia became the first country to introduce standardised packaging in December 2012, tobacco companies responded with more evocative and descriptive tobacco product names, including colours to represent the previous pack colour, thereby continuing the connotations associated with these colours(4, 5) . Simultaneously companies reduced the total number of brands sold and renewed their focus on value for money brands by increasing the number and length of cigarettes in a pack and introducing menthol variants in this price range.(6)

While the UK and EU legislation combined closed some of loopholes in the Australian legislation, by prohibiting evocative names and requiring minimum pack sizes for both cigarettes and RYO,(7) concerns remain. The current legislation does not permits the use of colour descriptors, bevelled edges on packs and cigarette filter technology innovations. Furthermore, cigars, cigarillos and pipe tobacco are exempt from the legislation and are still

For peer review only

For peer review only - http://bmjopen.bmj.com/site/about/guidelines.xhtml

sold in branded packaging, while wholesaler multipacks are still allowed branded 'outer' wraps.

Recently published research suggests that, prior to the full implementation of standardised packaging in the UK, tobacco companies added smaller pack sizes such as 17 sticks to their brand variant portfolios,(8) introduced colour descriptors to brand variant names and made product changes, particularly to filters.(9) Examples of name change patterns associated with UK standardised packaging restrictions have been described qualitatively(7, 9, 10) but not quantitatively. The scale of these name changes and if/how these changes were communicated to the public is not clear from published research. Previous work has also only explored up to two months post-legislation.

Therefore, the purpose of this paper was to use mixed methods to combine data from four different data sources up to 7 months post full-implementation to systematically and rigorously examine how compliant tobacco companies were with the legislation, and to explore any attempts they made to circumvent. We assess how their marketing has adapted including, changes to products or packaging that would endure after the legislation was fully implemented, and whether and how products are being marketed to retailers and consumers. The findings of this paper have global significance as they can help close loopholes during the design and implementation of standardised packaging legislation in other jurisdictions.

BOX 1. Summary of restrictions UK Standardised packaging legislation and the European Union Tobacco Products Directive Revision (implemented 20 May 2016 – 20 May 2017)

UK STANDARDARDISED PACKAGING	EU TOBACCO PRODUCTS DIRECTIVE

For peer review only - http://bmjopen.bmj.com/site/about/guidelines.xhtml

PACK EXTERNAL COLOUR: External packaging of cigarettes or RYO tobacco is Pantone 448C (matt finish)	PACKAGING OPENING: A unit packet of cigarettes may consist of carton or soft material and shall not have an opening that can be reclosed or re-sealed after it is first opened, other than the flip-top lid and shoulder box with a hinged lid. For packets with a flip-top lid and hinged lid, the lid shall be hinged only at the back of the unit packet.
PACK INTERNAL COLOUR: Internal colour of cigarette or RYO packaging must be white or Pantone 448C (matt finish)	PACK SIZE: Cigarette packs must contain a minimum of 20 cigarettes & RYO tobacco must contain at least 30 grams of tobacco
NOISE AND SMELL: Packaging must not make a noise or produce a smell that is not normally associated with the packaging	IMPRESSION CREATION: There must be no packaging elements that create an erroneous impression about the characteristics, health effects, risks or emissions of tobacco
PACKAGING EVOLUTION: The packaging must not change after retail sale, for example, heat activated inks, embellishments designed to appear gradually over time, scratch panels etc.	TOBACCO INGREDIENTS: No description of nicotine, tar or carbon monoxide content of a tobacco product

For peer review only - http://bmjopen.bmj.com/site/about/guidelines.xhtml

PACKAGING SHAPE: Cigarette packets must be made of carton or soft material and be cuboid in shape & RYO may be in a cuboid box, a cylinder or a pouch (bevelled or rounded edges are permitted)	FLAVOUR: No reference to taste, smell or any flavourings or additives or the absence of any such thing
STICK DESIGN: Cigarette stick paper must be plain white (matt finish), the filter must only be coloured in such a way as to imitate cork. The stick may have the brand name and variant printed in black Helvetica type no larger than 8 point in normal weighted regular typeface not more than 38 mm from the filter end of the cigarette. The start of each word can be an upper case letter but the rest of the word must be lower-case	ENVIRONMENT: No suggestion that a particular tobacco product has improved biodegradability or other environmental advantages
	RESEMBLANCE: No resemblance to a food or cosmetic product
	PROMOTION: No suggestion of economic advantage of one product over another – no price marked packs

For peer review only

For peer review only - http://bmjopen.bmj.com/site/about/guidelines.xhtml

Methods

Data Sources

We utilised four data sources, 1) purchases of eight top-selling tobacco brands; 2) tobacco adverts in retail trade publications; 3) other commercial literature: retail press articles and advertorials, tobacco company annual reports and Euromonitor market analyst reports; and 4) Nielsen data. While compliance with the legislation was primarily assessed using the Nielsen data, the evidence collected from each data source was utilised to assess whether, and how, tobacco companies adapted to the legislation and continued to market their products. We triangulated data from these different datasets not only to verify the existence of adaptations but also, their purpose.

Pack Purchasing and observation

We conducted monthly pack purchases between March 2016 and May 2017 to assess visual and sensory changes over time in the leading FM and RYO products (Table 1) at price points previously identified.(8)

Additionally, we observed price lists of cigar and pipe tobacco brands available in-store and the appearance of 'outers' (the cover wrap used to bind multiple packs together for distribution) where visible.

For peer review only - http://bmjopen.bmj.com/site/about/guidelines.xhtml

Table 1. Pack purchases of the top selling brand in each price segment

Price segment (average price per pack/stick) [1] [ref Nielsen paper]	Top selling products		Co [4]
	March 2016 name & pack size	May 2017 equivalent name & pack size	
FM			
Premium (£6.76/£0.42)	Marlboro Gold (20s)	Marlboro Gold (20s)	PMI
Midprice [2] (£5.61/£0.36)	L&B Original Lambert & Butler Silver (20s)<br>Mayfair King Size (19s)	Lambert & Butler Original Silver (20s)<br>Mayfair King Size (20s)	IMT<br>JTI
Value (£5.10/£0.33)	L&B Blue Lambert & Butler Real Blue (19s)	L&B Blue Real Blue(20s)	IMT
Subvalue (£4.68/£0.29)	Carlton Red (19s)<br>Rothmans of London[3] (17s)	Carlton Red (20s)<br>Rothmans Blue (20s)	IMT<br>BAT
RYO[5]			
Premium (£6.97/£0.17)	Golden Virginia The Original (25g)	Golden Virginia The Original (30g)	IMT
Midprice (£5.81/£0.15)	Amber Leaf (25g)	Amber Leaf Original (30g)	JTI

[1] weighted average price per pack/stick of all products sold within tobacco industry price segments as described by previous analysis (Dec 2015 prices – inflation adjusted to 2008 values). One RYO stick was estimated to be 0.5g tobacco.(8, 11)

For peer review only - http://bmjopen.bmj.com/site/about/guidelines.xhtml

For peer review only

[2]Midprice was originally two major segments: Lambert & Butler was the leading upper midprice product and Mayfair the leading lower midprice product

[3] None of the top selling brands in each price range was owned by British American Tobacco. For completeness we added Rothman's of London (a subvalue brand) - the top selling brand for British American Tobacco.

[4]Tobacco company' abbreviations: BAT British American Tobacco, IMT Imperial Tobacco, JTI Japan Tobacco International, PMI Philip Morris International

[5] No RYO value segment (average pack/stick price: £4.28/£0.14) product was purchased due to lower sales volumes even of the leading brand (Gold Leaf) in this segment.

Advertisements in the retail press

We searched hard copies of retail and wholesale publications *The Grocer, Wholesale News* and *Retail Newsagent* between January 2015 and December 2017 for all tobacco adverts.

We coded 195 adverts for the attributes marketed to retailers, eg, new brand variant, packaging, filter, limited edition, price, retailer profit. We examined the distribution of these marketing themes by price segment (Table S1). We separately assessed advertorials for relevance to standardised packaging and noted the main messages communicated.

Commercial literature review

We searched online retail publications (*The Grocer, Wholesale News, betterretailing.com* (which includes *Retail Newsagent* and *Retail Express, talkingretail.com*), to identify articles relevant to tobacco packaging and marketing (Jan 2015 – Dec 2017; Table 2). We also included two reviews of 2017 published in 2018, Euromonitor reports on tobacco, and tobacco company annual reports covering this period. We used NVivo 10, to code articles for any evidence of circumvention of the legislation and used quotes taken from the commercial literature as examples of each circumvention (Table S2).

For peer review only - http://bmjopen.bmj.com/site/about/guidelines.xhtml

For peer review only

Table 2 Commercial literature review January 2015 to December 2017

Source	*Search terms (depends on search procedure of website)*	*Total available*	*Total examined*
**RETAILER MAGAZINES**			
**Convenience & independents**			
Betterretailing.com: Retail Newsagent Retail Express	"tobacco"	350	181
TalkingRetail.com	"tobacco"	50	49
**Wholesalers &FMCG**			
Wholesale News	"tobacco" or "cigarette" or "ryo" or "roll+your+own" or "hand+rolled" or "cigar" or "cigarillo"	54	30
The Grocer	"tobacco" or "cigarette" or "ryo" or "roll+your+own" or "hand+rolled" or "cigar" or "cigarillo"	820	104
**INDUSTRY ANALYST**			
Euromonitor Passport	"tobacco"	NA	20
**TOBACCO COMPANY**			
Annual reports from the big four tobacco companies: JTI, IMT, BAT and PMI	Annual reports	12	12
**TOTAL**		1286	396

Nielsen Data

For peer review only - http://bmjopen.bmj.com/site/about/guidelines.xhtml

Nielsen collates data on tobacco sales from nearly 90% of UK supermarkets and a stratified sample of 15% of convenience stores. (8) Using a hierarchical structure Nielsen records for each product, the tobacco brand, brand family, brand variant and then specific features of the pack denoted by a unique serial number known as a SKU (e.g. size, pricemarked) (8). In November 2017 there were 71 brands, 97 families, 241 variants, and 1022 SKUs (Table 3).

For peer review only

For peer review only - http://bmjopen.bmj.com/site/about/guidelines.xhtml

Table 3 Hierarchical brand architecture available in the Nielsen data

		N (UK market)	
**Hierarchy of Nielsen data**	**Example**	**July 2014**	**Nov 2017**
BRAND	Marlboro	44	71
BRAND FAMILY Products with the same brand name but sold at different price points	Marlboro Bright Leaf	114	97
BRAND VARIANT Product at same price point but with different length (e.g. superkings, kingsize), flavour or other characteristics	Marlboro Bright Leaf Platinum	282	241
Stock Keeping Unit (SKU) Each individual barcoded product including specific pack characteristics: pack size, whether the pack is price-marked, and, for FM but not RYO, whether standardised or branded	Marlboro Bright Leaf Platinum 10s multipack not price marked	930	1022

Data Analysis

Compliance

For peer review only - http://bmjopen.bmj.com/site/about/guidelines.xhtml

Nielsen sales data were used to examine compliance with the removal of branding, pricemarking and small pack sizes (Box 1), between July 2016 (when the first standardised packs first were sold(10) and November 2017. For FM, Nielsen identifies which packs are standardised and which branded. For RYO it does not. However, as 30g packs of RYO were negligible prior to standardised pack legislation RYO packs of 30g or more were treated as standardised.

Name changes

We identified the number, names and price segments of all brand variants whose name changed between July 2014 and November 2017. We coded and counted whether each name change was required for compliance with the legislation and recorded the type of name change eg colour added or adjective added.

For peer review only

For peer review only - http://bmjopen.bmj.com/site/about/guidelines.xhtml

Innovation targeting

To understand whether innovations were targeted at particular price segments, we compared the distribution of name changes and new variants in November 2017 with the distribution of price segments prior to the decision to implement legislation in July 2014. Differences in the number of variants were tested using chi-square tests and Fishers exact tests (when the expected number of cases was less than 5). To achieve sufficient numbers for analysis, price segments were merged into four categories: (1) FM premium and midprice, (2) FM value and subvalue, (3) RYO premium and midprice, (4) RYO value.

Patient and Public Involvement

KER and RH led a focus group with smokers and former smokers from the UKCTAS University of Nottingham Smokers Panel (now known as the Tobacco & Nicotine Discussion Group) in October 2017. The group were asked to discuss their thoughts on and experiences of the move to standardised packs.

Results

Four main strategies were identified from multiple data sources. We present the evidence for each in turn.

Strategy 1: Keep branded packets on the market as long as possible

Six months into the year-long sell-through period, 96% of FM and 82% RYO were still sold in branded packs (Figure 1 & table S3). Most FM (70%) and RYO (65%) packs switched to

For peer review only - http://bmjopen.bmj.com/site/about/guidelines.xhtml

standardised packs between January and April 2017. One month after full-implementation, 1% FM and 2% RYO were sold in non-compliant packs, declining to 1% FM and negligible RYO by November 2017.

The commercial literature suggests that tobacco companies produced greater volumes of their branded FM and RYO tobacco prior to the May 2016 manufacturing deadline to keep branded packaging on the market for as long as possible during the sell-through.(12) Tobacco companies offered cash incentives and promotions on branded packs to retailers to encourage sales and loyalty to their products. By early 2017 this was described as a price-war between the manufacturers (13, 14) . To allay retailers' concerns about being left with non-compliant stock after the deadline, tobacco companies offered to buy them back (Table S2; Strategy 1.3).(15)

Strategy 2: Maintaining brand variant differentiation through name changes

A third (35%; 123/ 353) of variants changed name between July 2014 and November 2017. Of those, less than half changed name to comply with legislation (42%, n=52/123) and most of these removed flavour names as the legislation required but substituted with other names, most often a colour (Table 4). Most notably, FM smooth became bright or sky blue, with RYO smooth changing to yellow and menthol for both FM and RYO changing to green.

Of the variants which changed name when it was not necessary for legislative compliance (N=71/123, 58%), colours were introduced for the first time as a substitute for the previous pack colour, eg. *Marlboro* with the red chevron on the pack became Marlboro Red. Similarly, to distinguish brand variants from one another and to continue previous brand variant

For peer review only - http://bmjopen.bmj.com/site/about/guidelines.xhtml

identities, other adjectives were added to brand names, most notably adjectives accompanied a colour, e.g. *real* blue, *bright* silver, *legendary* black. In addition, during the study period, 23 new variants appeared in the Nielsen dataset with names which included elements that went beyond the purpose of practical information: ‘signature’, ‘ome’, ‘silver strand’ in May 2015, ‘Eagle,’ and ‘Colours’’ in November 2015 and ‘Chill’ in May 2016 and two different brands added ‘Black Russian’ variants in November 2015 and January 2017. Lastly two new Golden Virginia RYO variants appeared on the market in November 2015 named ‘Midnight’ and ‘Sunrise’. Commercial literature revealed that these indicated rich and smooth flavours respectively. (16). The first brand variant in a brand family sometimes had ‘original’ added e.g. Amber Leaf RYO became Amber Leaf original.

For peer review only

For peer review only - http://bmjopen.bmj.com/site/about/guidelines.xhtml

Table 4. FM and RYO brand variant name changes (July 2014 to November 2017)

		N	Additions/substitutions
**Legally required actions**			
Taste removed	*Replacement*		
Menthol	Colour	20	green
	No replacement	2	
Smooth	Colour + adjective	12	bright blue/yellow, sky blue
	Adjective	5	bright, sky
	Colour	3	blue, yellow
	Other Substitution	1	fine
other taste ('fresh burst', 'fresh taste', 'ice capsule', 'subtle flavour')	Other Substitution	3	crushball, dual
	No replacement	1	
Environmental advantages removed			
'Natural'	Other Substitution	2	blue, king size
	No replacement	3	
Total		52	
**Discretionary actions only**			
colour+adjective added		9	original silver/blue/black, real blue/red
adjective added		8	bright, real, legendary
colour added		7	blue, red, black
'original' added		6	
size (king size, superkings) removed		6	
Mixed & miscellaneous		35	

For peer review only - http://bmjopen.bmj.com/site/about/guidelines.xhtml

Total	71
**Grand total**	**12<br>3**

In October 2016, five months into the sell-through period and before IMT had made any of its legally required name changes to its products, the company outlined its brand renaming strategy in an advertorial in the retail press.(17) The advertorial explained that all products traditionally presented as full flavour would become 'real' or 'original' and be associated primarily with the colour red. Those previously denoted as 'smooth' would change to 'bright' and become associated with the colour blue. Menthol would become green and capsule would become Crushball (Table S2: Strategy 2). IMT also provided retailers with materials to help explain changes to consumers.(17) Some companies also advertised name changes directly to consumers through pack inserts (eg. BAT's Rothmans of London included a pack insert to warn its smokers that it was to become Rothman's Blue).

Strategy 3. Focus on lower price segments

During the study period, 68% of new variants recorded by Nielsen were FM and 32% RYO. Given that in July 2014, 81% variants on the UK market were FM and 19% were RYO (Table 5), more new RYO variants were introduced than expected (p=.018).

Half (50%) of all new variants were introduced in the FM value and subvalue price segments, 22% FM premium and midprice, 15% RYO premium and midprice and 13% RYO value. However, prior to the decision to implement standardised packaging (July 2014), the distribution of the market was 50% FM premium and midprice, 35% FM value and subvalue,

For peer review only - http://bmjopen.bmj.com/site/about/guidelines.xhtml

8% RYO premium and midprice and 7% RYO value. Thus new variants were under-represented among FM premium and midprice brands (p=.001).

Similarly, compared with July 2014, name changes in the study period were overrepresented among FM value and subvalue (p=.048) and under-represented among FM premium and midprice brands (p=.002).

For peer review only

For peer review only - http://bmjopen.bmj.com/site/about/guidelines.xhtml

Table 5 Comparison of distribution of variants prior to the decision to enact legislation (July 2014), variants with name changes and new variants that entered the market.

	Baseline distribution of variants (July 2014)		Name changes (July 2014 – November)			New variants (November 2018)		
	N	%	N	%	P*	N	%	P*
**Tobacco Type**								
FM			12		.527			.018
	225	81%	5	79%		48	68%	
RYO	54	19%	35	21%		23	32%	
**Price segment**								
FM premium & midprice	103	50%	48	33%	.002	10	22%	.001
FM value & sub value	72	35%	66	45%	.048	23	50%	.054
RYO premium & midprice					.097			.164
	17	8%	20	14%		7	15%	#
RYO value					.735			.602
	15	7%	12	8%		6	13%	#

*Chi square comparing with July 2014 distribution

#Fishers exact test comparing with July 2014 distribution

Nearly two-thirds of the adverts captured from the retail press, 124/195 (64%) advertised value or subvalue brands. Similarly, for new products, the majority of adverts (30/38; 79%) were for value or subvalue brands. Only one advert was a new premium offering.

The commercial literature found tobacco companies innovating within lower price segments, introducing packs with fewer cigarettes prior to the sell-through (18-20) followed by a price-war in the lower priced segments with some manufacturers reportedly selling at a loss to gain market share.(13) By the end of 2017 tobacco companies stated that smokers expected better quality products in lower price segments after standardised packaging and Minimum Excise Tax (MET) legislation raised the price of the cheaper brands and so ‘premium features’ in lower priced cigarettes appeared (Table S2: Strategy 3).(19, 21) For example, a

For peer review only

For peer review only - http://bmjopen.bmj.com/site/about/guidelines.xhtml

redesign to Chesterfield (subvalue) was advertised to retailers in December 2017, citing a new ‘firm’ filter and, as with premium brand Marlboro, a new bevelled edge box.(20, 22)

For peer review only

For peer review only - http://bmjopen.bmj.com/site/about/guidelines.xhtml

Strategy 4. Innovating exemptions to the legislation

The commercial literature and pack purchase study suggested that tobacco companies continued to innovate and market their tobacco products by focusing on exemptions to the legislation (Table S2: Strategy 4).

Pack modifications that endured post-legislation

During the sell-through, the pack purchase and commercial literature review revealed that FM Marlboro 10s and RYO *Amber Leaf*, *Golden Virginia* and *Cutter's Choice* were sold in reusable tins, arguably allowing consumers to decant cigarettes bought post-legislation into branded tins. Selling RYO in tins is not novel but cigarettes have rarely been sold in tins previously.

Pack purchases found that best-selling premium brand family *Marlboro* changed significantly in July 2016 (two months into sell-through), with the introduction of bevelled pack edges and a *pro-seal* closing mechanism (Figure 2). Both features fundamentally changed the tactile nature of the pack which endured after full-implementation in May 2017 (Video 1). To be compliant with the legislation, these packs must have been printed prior to May 2016.

Extra Sticks

Given that there is no restriction on the maximum number of sticks per pack, packs with more than 20 continued post-legislation. Following full-implementation sales of packs with 23 and 24 sticks increased from 7 to 11 million packs.

Branded Outers

The retail literature and in person observations revealed that multipack wholesale outers are

For peer review only - http://bmjopen.bmj.com/site/about/guidelines.xhtml

For peer review only

branded (Figure 3). Outers can be seen by customers during tobacco product gantry restocking. Adverts in the retail press showed images of branded tobacco products including branded outers post-legislation.

RYO accessories

Increased innovation was observed among RYO accessories post legislation with retail press articles in November 2017 and February 2018 referring to "'ultra slim' and slim filters and papers, biodegradable filters, and menthol tips" (Table S2; Strategy 4.4). (21, 23)

Cigars

Cigars, which can be sold in branded packs, single sticks without pictorial health warnings, at a relatively low price and with good profit margin, were identified as an opportunity for growth.(20, 24-27) Euromonitor's models suggested growth in cigars and cigarillos sales volumes (390 to 494 milllion units) and value (£292 to £348 million) from 2015 to 18 (Table S4). Towards the end of 2017, JTI and cigar company Ritmeester were holding social events to build relationships with retailers (Table S2; Strategy 4.5). (20)

Pipe Tobacco

The retail literature revealed that one small tobacco manufacturer, *Gawith Hoggarth*, deliberately marketed pipe tobacco as RYO to circumvent the minimum pack size restrictions on RYO (Table S2; Strategy 4.6).(28)

Discussion

UK Standardised Packaging legislation and the EU TPD placed restrictions on tobacco packaging and marketing in the UK. Overall, compliance with the restrictions (removal of branding, small pack sizes and non-compliant names) was good. However, clarity is needed about the legality of isolated incidents such as the compliance of the *Marlboro* resealable

For peer review only - http://bmjopen.bmj.com/site/about/guidelines.xhtml

pack and Gawith Hoggath's marketing of its *Kendal* pipe tobacco as RYO. Technical compliance aside, this paper identified four key strategies of tobacco companies which have arguably enabled them to continue marketing tobacco post-legislation.

Firstly, the retail literature suggests that tobacco companies used the 12-month sell-through to their advantage, keeping branded stock on the market as long as possible, and using the time to communicate name changes to retailers and customers. Ahead of the sell-through companies increased production of branded packs, introduced smaller pack sizes to enable more affordable offerings, and encouraged retailers to buy large quantities of branded stock at reduced prices. Other countries had a shorter sell through period with three-months for Australia and New Zealand (29, 30) and seven months for France (31). Other countries considering the legislation should resist calls for a lengthy sell-through.

Second, although compliant with legally required name removals, tobacco companies implemented a standard name change formula that maintained the brand identity and differentiation of the three broad flavour categories (1) full flavour; (2) smooth; (3) menthol.(17) In 2002 terms such as 'mild' and 'light' were prohibited to curb misperceptions of relative harm. However, the continuation of the colours of the previous packs such as the gold and white pack for *Marlboro 'lights'* and the introduction of terms such as smooth for other brands sustained these misperceptions.(32, 33) In line with the power of colour in brand identity, this study shows that tobacco companies changed '*full-flavour'* variants to *red, original,* or *real; menthol* variants to *green*; and *smooth variants* to *bright* and *blue (*or *yellow* for RYO). As in 2002, the current restrictions on tobacco product name were designed disrupt misperceptions of relative harms based on flavour descriptions but yet again, tobacco companies have adapted to ensure that such misperceptions are likely to

For peer review only - http://bmjopen.bmj.com/site/about/guidelines.xhtml

endure. Therefore there is a case to be made to follow the example of Uruguay where only one brand variant is permitted per brand and no new brands are allowed.

Third, Nielsen data and the commercial literature revealed that tobacco companies were fighting fervently for market share in the cheaper price segments with a price war pre-implementation, more adverts, name changes, extra sticks in packs and new brand variants introduced in lower price segments over the study period. This taken in concert with our fourth finding that tobacco companies are targeting tobacco products, features and accessories not covered by the UK or EU legislation for innovation and growth provides a case for expanding the legislation to include: standardised packaging for cigars, cigarillos, pipe tobacco, wholesale outer wraps, standardised pack edges, maximum pack sizes and prohibitions for innovations to filters, pack seals RYO papers.

The strength of this paper lies both in the detail and depth of each analysis including our systematic analysis of retail press adverts (n=195) and commercial literature articles and reports (n= 396) alongside pack purchases of the top selling brands and detailed sales information from Nielsen. Using multiple data sources has, in many instances, enabled findings to be verified by more than one source and enabled a greater understanding of the tobacco industry's motives for any changes made to their products and packaging. Nevertheless, despite evidence from an IMT whistleblower(34) and PMI's own words(35) our data did not reveal price mark stickers on tobacco products, this is likely to be because we bought our eight brands from a large supermarket and not a convenience store and because this industry strategy was not highlighted in the retail literature due to the questionable legality of this strategy.(36)

For peer review only - http://bmjopen.bmj.com/site/about/guidelines.xhtml

Resources prevented us from acquiring Nielsen data on cigars or pipe tobacco and from purchasing more than one brand per price segment in the pack purchasing study element. However, the retail literature alerted us that cigars and other products will be targeted as growth opportunities and, although not able to fully capture the sensory nature of brands, our analysis of the adverts ensured that we would see many, if not all, innovations being promoted to retailers.

Nielsen data does not record whether RYO products are sold in standardised packs. We assumed that 30g packs were always in standardised packs and that larger packs would switch to standardised packs at the same time. It is possible that this did not occur. However the temporal patterns of switching names and sizes are similar for FM and RYO and it is therefore reasonable to assume that branding was removed at the same time. Nielsen model prices and volumes for the UK based on a census of the major supermarket sales and a rolling sample of convenience stores. Although, we do not know the extent to which Nielsen variant name changes lag behind and even reflect those printed on packs in retailers, the main name change patterns found in the Nielsen data were similar to those found in a UK convenience store study(37) and an evaluation of the introduction of standardised packaging in Australia.

Given that the tobacco industry is attempting to circumvent standardised packaging legislation, it is incumbent on other countries considering the policy to make regulations as comprehensive as possible to prevent the exploitation of continued marketing opportunities. In summary, this paper suggests that long sell-through periods should be avoided and that restricting tobacco products to one brand variant per brand and an extending regulations to include other tobacco products, features and accessories should be considered.

For peer review only - http://bmjopen.bmj.com/site/about/guidelines.xhtml

For peer review only

For peer review only

For peer review only - http://bmjopen.bmj.com/site/about/guidelines.xhtml

Author contributions: AG designed the study with contributions from KER and RH. KER led the writing of the paper and designed, conducted and analysed the pack purchase element of the study. RH analysed the Nielsen data in addition to downloading and analysing the commercial literature. KL collated the retail literature adverts, involving visits to the British Library and analysed the data. All authors read and approved the manuscript. The authors would like to thank Dr Rob Branston who read and commented upon a near final draft and Ilhan Marsal who helped with early preparation of the adverts and pack purchased data.

Data statement: Where suitable data sets have deposited in the University of Bath data archive.

For peer review only

For peer review only - http://bmjopen.bmj.com/site/about/guidelines.xhtml

References

1. legislation.gov.uk. The standardised packaging of tobacco products regulations. In: Uk Government, editor. 829; 2015.
2. Department of Health. Tobacco packaging guidance, guidance for retailers, manufacturers and distributors of tobacco products, enforcement agencies and the public on changes to tobacco packaging from 20 may 2016. Guidance; 2016.
3. Official Journal of the European Union. Directive 2014/40/eu of the european parliament and of the council of 3 april 2014. In: European Union, editor.; 2014.
4. Scollo M, Occleston J, Bayly M, Lindorff K, Wakefield M. Tobacco product developments coinciding with the implementation of plain packaging in australia. Tobacco Control. 2014 April 30, 2014.
5. Scollo M, Bayly M, White S, Lindorff K, Wakefield M. Tobacco product developments in the australian market in the 4 years following plain packaging. Tobacco Control. 2017.
6. Scollo M, Zacher M, Coomber K, Bayly M, Wakefield M. Changes in use of types of tobacco products by pack sizes and price segments, prices paid and consumption following the introduction of plain packaging in australia. Tobacco Control. 2015 April 1, 2015;24(Suppl 2):ii66-ii75.
7. Moodie C, Hoek J, Scheffels J, Gallopel-Morvan K, Lindorff K. Plain packaging: Legislative differences in australia, france, the uk, new zealand and norway, and options for strengthening regulations. Tob Control. 2018 Aug 1.
8. Hiscock R, Branston JR, McNeill A, Hitchman SC, Partos TR, Gilmore AB. Tobacco industry strategies undermine government tax policy: Evidence from commercial data. Tobacco Control. 2017;27:488-97.
9. Moodie C, Angus K, Mitchell D, Critchlow N. How tobacco comapnies in the uk prepared for and responded to standardised packaging of cigarettes and rolling tobacco. Tobacco Control 2018.
10. Breton MO, Britton J, Huang Y, Bogdanovica I. Cigarette brand diversity and price changes during the implementation of plain packaging in the united kingdom. Addiction. 2018.
11. Branston JR, McNeill A, Gilmore AB, Hiscock R, Partos TR. Keeping smoking affordable in higher tax environments via smoking thinner roll-your-own cigarettes: Findings from the international tobacco control four country survey 2006–15. Drug and Alcohol Dependence. 2018;193:110-16.
12. Retail Newsagent. Explained: Retailer's questions about eutpd ii. Retail Newsagent; 10 May 2016.
13. BetterRetailing.com. Value brands price war hits retail profits. Retail Express 17 February 2017 [cited; Available from: https://www.betterretailing.com/value-brands-price-war-hits-retail-profits
14. Retail Newsagent. Cash incentives and price cuts drive tobacco sales. Retail Newsagent; 26 May 2016.
15. Samrai G. Wholesalers: Tobacco suppliers will help retailers with branded stock post-eutpd ii. Retail Newsagent; 2 March 2017.
16. Imperial tobacco unveils gv midnight and sunrise. Talking Retail; 17 July 2015.
17. Imperial Tobacco. Partnering for success through eutpd ii and standardised packaging: Part 4. New pack descriptors under eutpd ii. Retail Newsagent. 28 October 2016:20-21.
18. O'Donnell T. The fat lady sings: Tobacco category report 2016. The Grocer; 11 February 2016.
19. Selwood D. Pall mall cigarettes get taste plus filter in range upgrade. The Grocer; 18 April 2016.
20. Yau A. The latest developments from the biggest tobacco brands. Retail Newsagent; 5 April 2018.
21. Pendleton R. A tightening stranglehold: Tobacco category report 2018. The Grocer; 9 February 2018.

For peer review only - http://bmjopen.bmj.com/site/about/guidelines.xhtml

22. Philip Morris International. Stock up now: Chesterfield with new premium features. Retail Newsagent. 15 December 2017.
23. Cronin E. Rizla launches new natura paper and tips range. Talking Retail; 3 August 2017.
24. Appleby S. New tobacco law, means new opportunity for cigars sales. Retail Express; 6 June 2016.
25. Dillon C. Three things you can learn from ritmeester's cigar seminars. Retail Express; 17 September 2017.
26. Dillon C. Ritmeester launches single stick panatellas. Retail Express; 15 January 2015.
27. Lee J. Know the value of the tobacco market to keep sales strong. Retail Express; 22 August 2017.
28. Gockelen-Kozlowski T. Tobacco firms adapt to deal with latest eutpd laws. Retail Newsagent; 25 May 2017.
29. Ministry of Health (New Zealand). Tobacco standardised packaging. 2017 13th June 2017 [cited; Available from: https://www.health.govt.nz/our-work/preventative-health-wellness/tobacco-control/plain-packaging
30. Scollo M, Lindorff K, Coomber K, Bayly M, Wakefield M. Standardised packaging and new enlarged graphic health warnings for tobacco products in australia—legislative requirements and implementation of the tobacco plain packaging act 2011 and the competition and consumer (tobacco) information standard, <em>2011</em&gt. Tobacco Control. 2015;24(Suppl 2):ii9.
31. Tobacco Labelling Resource Centre. France. 2013 [cited; Available from: http://www.tobaccolabels.ca/countries/france/
32. Mutti S, Hammond D, Borland R, Cummings MK, O'Connor RJ, Fong GT. Beyond light and mild: Cigarette brand descriptors and perceptions of risk in the international tobacco control (itc) four country survey. Addiction. 2011;106(6):1166-1175.
33. Bansal-Travers M, O'Connor RJ, Fix BV, Cummings MK. What do cigarette pack colors communicate to smokers in the u.S.? American Journal of Preventive Medicine. 2011;40(6):683-89.
34. Doward J. How tobacco firms flout uk law on plain packaging. 9 April 2017 [cited 12 November 2018]; Available from: https://www.theguardian.com/society/2017/apr/09/tobacco-companies-flout-law-plain-packaging
35. Nixon P. Re: Price marking on packs. Letter from Peter Nixon, Philip Morris International to Deborah Arnott, Action for Smoking and Health ed; 15 May 2017.
36. Leigh Day. Re: Price marking on tobacco packaging. Legal opinion of Leigh Day to Action for Smoking and Health ed; 3 March 2017.
37. Critchlow N, Stead M, Moodie C, Eadie D, MacKintosh AM. Introduction of standardized tobacco packaging during a 12-month tranistion period: Findings from small retailers in the united kingdom. Nicotine & Tobacco research. 2018.
38. Yau A. Premium features sell cigarettes. Retail Newsagent; 4 December 2017.
39. Euromonitor. Passport: Philip morris intenrational inc in tobacco (world). July 2017.
40. Cronin E. Imperial tobacco unveils new innovations for 2018. Talking Retail; 24 November 2017.
41. Marlboro launches ducati promotion. Talking Retail; 31 July 2015.
42. Whitlock K. Imperial tobacco launches jps triple flow. Wholesale News. November 2015.

For peer review only - http://bmjopen.bmj.com/site/about/guidelines.xhtml

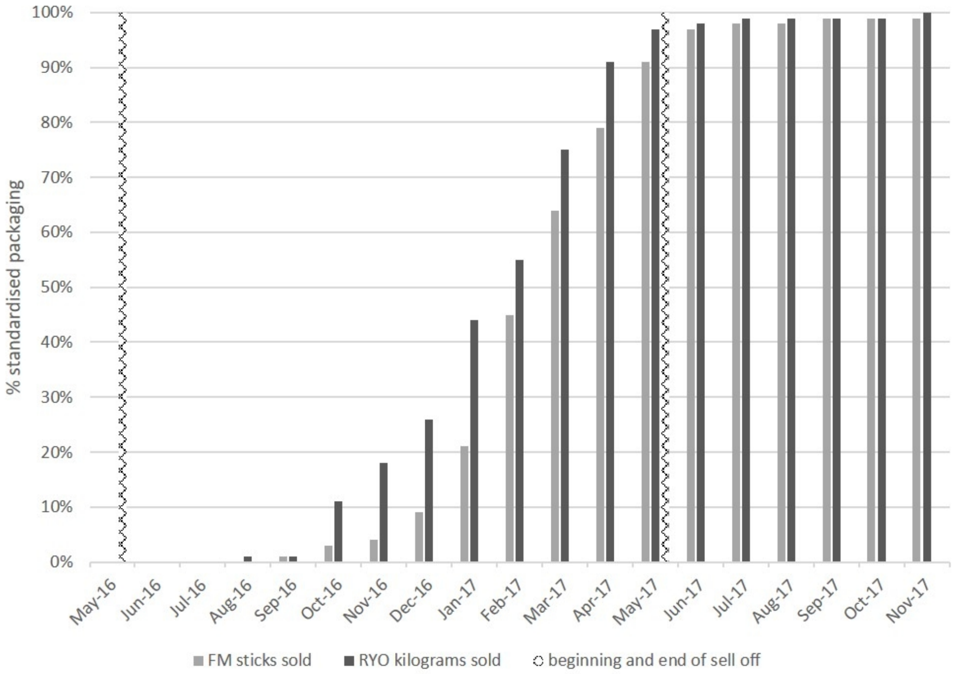

Figure 2. Proportion of (a) FM sticks and (b) RYO* (by weight) sold in standardised packaging over time

85x60mm (300 x 300 DPI)

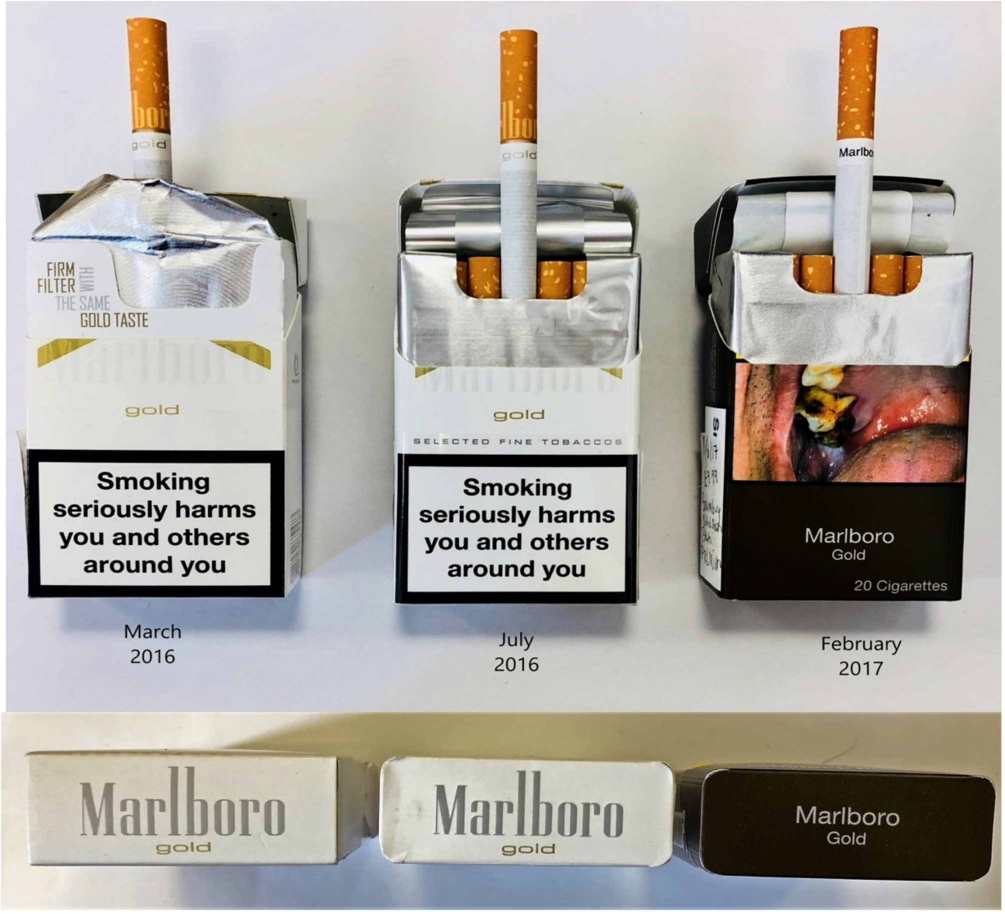

Figure 3. The evolution of Marlboro Gold packaging from a straight edge pack to a branded bevelled edge pack with a new internal packet with pro-seal sealing mechanism, to standardised packaging maintaining these innovations

103x94mm (300 x 300 DPI)

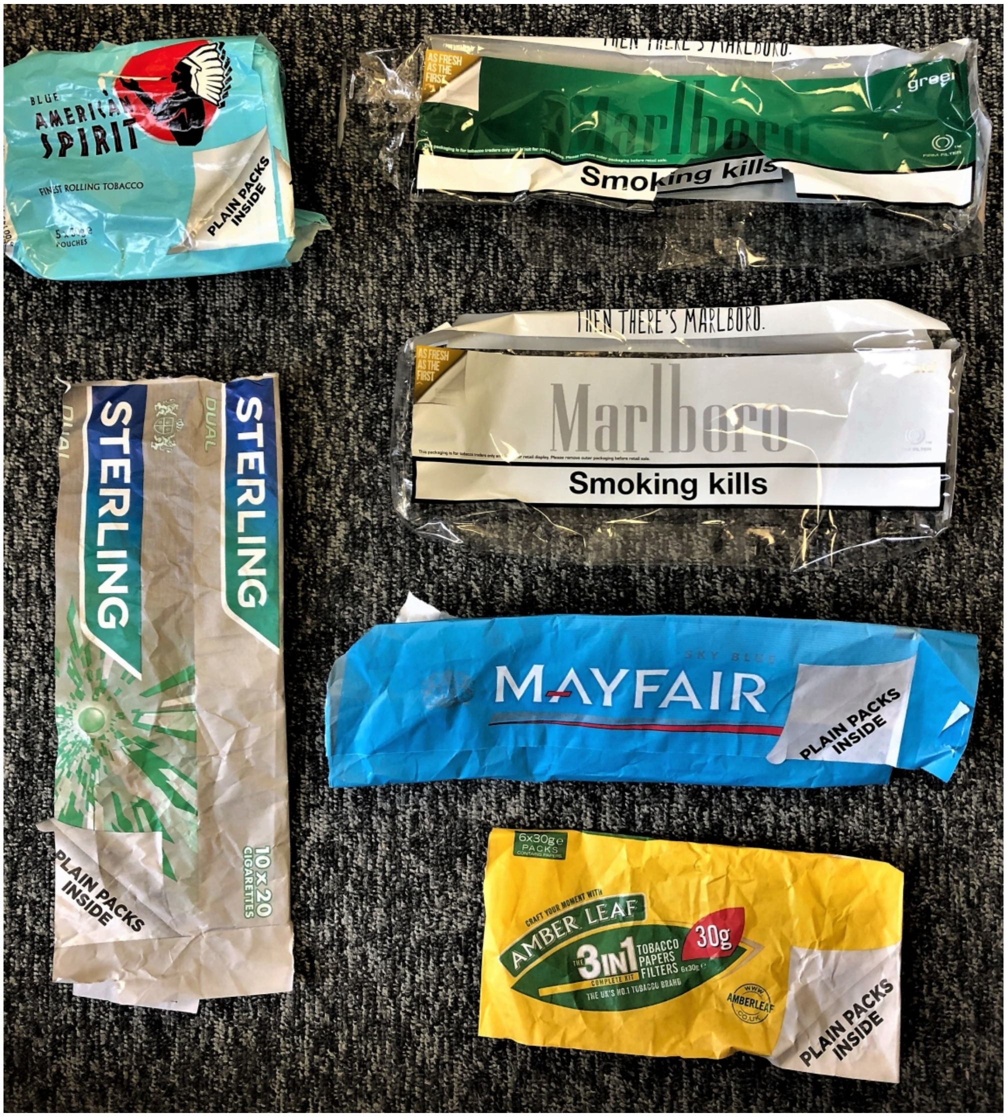

Figure 4. Branded outers post May 2016 (full-implementation)

256x283mm (300 x 300 DPI)

Supplementary file material for "Assessing tobacco company adaptation to standardised packaging: identifying circumventions and closing loopholes"

Table S1. Tobacco advert characteristics by price segment (n= 195) [1]

Characteristics of advert[2]	Subvalue[3]	Value	Midprice	Premium
Type of tobacco				
FM	22	78	42	21
RYO	n/a	24	13	16
Characteristics promoted				
New brand variant[4]	7	23	5	1
New packaging	4	25	17	16
Limited Edition	0	4	5	5
Filter	0	16	0	6
Price	19	68	14	4
Retailer profit	1	42	21	5

[1] Totals do not add up to 195 adverts as many adverts promoted more than one type of tobacco, more than one price segment and more than one characteristic in the same advert.

[2] Does not include pipe tobacco or cigars/cigarillos as we have no data on price segment for these products.

[3] FM only. This price segment does not exist for RYO.

[4] There were 38 adverts in total for a new brand variant yet 2 of the adverts could not be categorised by price segment.

Characteristics promoted	Cigars/ cigarillos	Pipe
New brand variant	0	0
New packaging	0	0
Limited Edition	2	0
Filter	0	0
Price	2	1
Retailer profit	6	1

For peer review only - http://bmjopen.bmj.com/site/about/guidelines.xhtml

For peer review only

Table S2 Industry strategies to circumvent standardised packaging and exploit loopholes implied by commercial literature

Industry strategy	Tactic	Quote from the commercial literature
1. Keep branded packaging on the market throughout the sell-off period	1.1 Over-producing branded packs before the sell-off	"BAT has had to produce higher stocks prior to the 20 May 2016 manufacturing deadline in order to avoid out of stocks [branded]…The TPD explicitly provides for transitional provisions to allow product manufactured before 20 May 2016 to be sold until 20 May 2017." Ron Ridderbeek, head of legal and external affairs at British American Tobacco [*Retail Newsagent*, 10 May 2016]
	1.2 Introducing smaller pack sizes just before the sell-off	"Tinkering with pack sizes is a proven wheeze for off setting the impact of tobacco's growing tax burden on price per pack. JTI has managed to hit the £6.50 price point by launching 17-packs of Sovereign Blue, Sky Blue, Green and Dual. And it's just in time; from May, pack sizes will be limited to a minimum of 20 cigs." Japan Tobacco International [*The Grocer,* 11 February 2016]<br><br>"Launching next month as the European Union Tobacco Productive Directive comes into force, [Pall Mall's] redesign - available in 10-pack and 18-pack King Size and Super King 18s - will include the new Taste Plus Filter … price-marked packs are set to be available at the current [price]." [*The Grocer,* 18 April 2016]
	1.3 Providing financial incentives to retailers to sell a particular company's brands	"Retailers have reported a "massive" increase in cigarette sales as tobacco manufacturers cut prices and offer cash incentives to bolster brands at a time when new plain packaging and smaller pack regulations are set to shake up the industry. … An independent retailer… said: "I have seen a massive increase in sales. We were also given PoS including shelf wobblers to encourage as many customers as possible to buy the brand. I think this is a really smart way of driving sales and is something that retailers will respond to." Meanwhile, Imperial Tobacco will knock 10p off the price of four of its biggest tobacco brands - Lambert & Butler, JPS, Players and Gold Leaf - from 1 June, while maintaining cash margins... [A retailer who] has been trialling the new Imperial prices at his store… said: "We sold up to one and a half extra outers a week because of the lower price." [*Retail Newsagent,* 26 May 2016]<br><br>"A value brands price war on cigarettes is hurting retailers' margins and could lead to stores being "lumbered" with a heap of unwanted, unsaleable stock [with legislation changes;] tobacco suppliers have been dropping the prices of cigarette packs at the value end of the market… some manufacturers… selling value cigarettes at a loss to gain market share… Last runs of branded packs are price-marked lowly and [a retailer] told Retail Express that the actions of suppliers will have grave consequences for unsuspecting retailers.<br><br>"Situations will arise where retailers are taken in by the low, price-marked RRPs and buy in bulk, thinking that consumers will jump at the chance to catch the last batches of cheap packs…Come May 20, they'll find themselves lumbered with packs of cigarettes they can no longer sell, as supply will outstrip demand…It's a

For peer review only - http://bmjopen.bmj.com/site/about/guidelines.xhtml

		unique situation, but the suppliers have stock they need to get rid of. They're cutting the prices down and loss-leading on them [but]Independent retailers certainly can't afford to loss-lead. "…[Another retailer] said: "… Companies downtrading to push their own agendas does not help us at all. I've even heard that certain wholesalers were offered better percentages to buy in these packs, which would obviously be recouped at the other end.""[*Better Retailing,* 17 February 2017]<br><br>""The tobacco suppliers will themselves take back any branded packs after the May cut-off," [wholesaler manager] told RN. "They have teams of people calling on retailers and they are notifying them as to the process. They are saying if they have any branded stock left to call their local rep and they will come in and replace it." [*Retail Newsagent*, 2 March 2017]
2. Maintain brand differentiation through systematic name changes		"We recognise that consumers use brand and colour when asking for their pack of cigarettes." Therefore, generally, those products traditionally presented as 'full flavour' would have 'real' or 'original' added to their names and be denoted predominantly by the colour 'red'. All brand variants with the flavour descriptor 'smooth' would change to 'bright' and have 'blue' added, 'menthol' would become 'green' and 'capsule' would become 'crushball'." [*Retail Newsagent*, 28 October 2016]
3. Focusing on lower price segments	Introducing premium features into cheaper price segments	"Living in a post-EUTPD II world… [a retailer reported] "Customers have become more price-sensitive since legislation banned the sale of 10- packs and they are more concerned about how much they can get for their money these days"…To help retailers tap into the trend for budget tobacco, JTI has relaunched Scottish brand Kensitas Club King Size and dropped its RRP " Yau A. The latest developments from the biggest tobacco brands. [*Retail Newsagent*, 5 April 2018]
4. Promote products by innovating exemptions to the legislation	4.1 Bevelled edges and resealable packs	"In order to re-engage disengaged cash-strapped smokers migrating to lower-priced brands and categories, manufacturers have been "adding value" to their brands through innovation, both on the pack and via the product itself… PMI concentrated on areas such as pack size (extra-large pack sizes), pack type (resealable), cigarette length (compact), microslims, filter type (with a focus on recessed filters) and flavour capsules (including the world's first recessed filter capsule and the world's first double capsule – menthol and peppermint). Pack innovation: Tactile finishes, including raised surfaces such as embossing and bevelled edges… (39)[1] [*Euromonitor*, July 2016]
	4.2 Menthol	"JPS Green Edge will replace it and feature a menthol inner liner within the packaging, which the company claims will be "more effective at transferring flavour to the cigarettes than traditional menthol tips"". Imperial Tobacco [*Talking Retail*, 24 November 2017]

[1] This Euromonitor report was published two months into the sell-off period. We did find evidence of embossing and raised surfaces on packets prior to the full implementation of the legislation but these changes were lost when standardised packaging came into effect. During the sell-off, filter innovations, capsule innovations, bevelled edges and a pack resealing mechanism on one brand were observed – these changes were able to carry through when packs became standardised. In this study we did not observe extra length cigarettes, a greater volume of cigarettes or microslims.

For peer review only - http://bmjopen.bmj.com/site/about/guidelines.xhtml

	4.3 Filters (another way to create the perception that some products are healthier than others?)	Marlboro Touch, previously known as Marlboro Gold Touch, now has a soft touch pack and firm filter keeping the consistency with the rest of the Marlboro range. The firm filter allows adult smokers a cleaner way to stub out their cigarette, as well as adding an overall feeling of quality, without having an impact upon taste." [*Talking Retail,* 31 July 2015]<br><br>"Launching …as the European Union Tobacco Productive Directive comes into force, [Pall Mall's] redesign… will include the new Taste Plus Filter that aims to create a firmer in-hand feel and improve filtration" [*The Grocer,* 18 April 2016]<br><br>"…JPS Triple Flow… "a revolution in cigarette technology." Triple Flow uses "unique" "easy draw" channels, with a new, "high quality smooth" tobacco blend enhanced by a mineral filter that provides "a fuller smoking experience". Each cigarette is also wrapped in a special paper, designed to actively reduce the amount of smoke emitted - which may result in less smell, says Imperial. Brand portfolio manager Emma Stew said: "Smooth blends now account for almost a quarter [ITUK estimates September 2015] of factory made cigarette (FMC) sales, and Triple Flow has been designed to offer the ultimate smooth smoking experience. 'With its next generation features and distinctive packaging, there is nothing like it in the market'" [*Wholesale News* November 2015]
	4.4 RYO papers and filter tips	Imperial Tobacco brand manager Jamie Burns-Smith said: "Natura taps into an increasing consumer desire for responsibly sourced products. Crafted from 100% hemp, Natura papers and tips are the perfect sustainable way to enjoy an authentic tobacco taste. Imperial Tobacco has also launched Rizla Menthol Tips, as the company claims "menthol tips are the fastest-growing segment of the tips market". [*Talking Retail,* 3 August 2017]
	4.5 Cigars	"Break, as they are cigarillos, are exempt. We can do packs of less than 20, and we can do flavours… Cigars won't be a standard colour pack, and will still have product name branding… you can sell individually and in 10 packs" Jens Christiansen, Scandinavian Tobacco Group's head of marketing & public affairs [*Retail Express*, 6 June 2016]<br><br>"Cigars benefit from the fact they can be sold in 10-packs, while also being exempt from certain branding restrictions. Scandinavian Tobacco Group (STG) is using this to its advantage and its marketing this year… the packaging stands out when compared to traditional cigarettes… and the investment made by companies such as STG to raise awareness and education in the convenience trade really helps... Ritmeester's says miniature cigars have traditionally been products that are better known among tobacco enthusiasts. As a result, it is offering samples of its Royal Dutch range to retailers to increase awareness…

For peer review only - http://bmjopen.bmj.com/site/about/guidelines.xhtml

		"I find the curry socials Ritmeester holds with retailers and the trade press very useful. It's a hands-on, but informal way of learning about the latest developments in the cigar market. There isn't as much awareness about cigars as cigarettes. These opportunities give us a chance to learn about how we can upsell to smokers who want value." [Retailer]" [*Retail Newsagent*, 5 April 2018]<br><br>"If a smoker only has £5 on them, they cannot buy from your gantry, but if you present them with cigars they can." Ritmeester [*Retail Express*, 17 September 2017]<br><br>"[Retailer] says he struggles with selling Mini Moods. [Ritmeester's] Lyles explains: "It might be because you're aiming them at cigar smokers, but it's primarily aimed at cigarette smokers. Because of the double filter, it makes for a smoother smoke." … Cigars are now the cheapest product in a gantry. Lyles says: "If a smoker only has £5 on them, they cannot buy from your gantry, but if you present them with cigars they can." [*Retail Express*, 17 September 2017]<br><br>"'The margins we make on miniature cigars are 30% when sold at RRP' [Retailer]" [*Retail Newsagent*, 5 April 2018]
	4.6 Pipe tobacco sold as RYO	"The sell-through period for branded RYO and cigarettes ended last week, with products now sold in minimum formats and carrying larger health warnings as part of the new tobacco legislation. Yet, UK tobacco manufacturer Gawith Hoggarth is promoting its Kendal brand as a product which is cut to pipe tobacco regulations but can also be used as hand-rolling tobacco. "We've cut the tobacco to the thinnest width we are allowed to which means we can sell it in branded packaging and in 12.5g pouches," said the company's area sales manager Martin Bobbette. The company hopes retailers will recommend the product to smokers who ask for "whatever is cheapest"" [*Retail Newsagent*, 25 May 2017]

For peer review only - http://bmjopen.bmj.com/site/about/guidelines.xhtml

Table S3 Compliance with standardised packaging over time[1]

	% standardised packs	change from previous month	total sticks sold	non-compliant sticks sold
Jul-16	0%		2,574,340,308	2,574,204,015
Aug-16	0%	0%	2,620,104,622	2,610,727,340
Sep-16	1%	1%	2,424,692,602	2,397,886,927
Oct-16	3%	2%	2,491,577,487	2,423,864,794
Nov-16	4%	1%	2,424,660,803	2,323,057,266
Dec-16	9%	4%	2,593,371,625	2,370,713,888
Jan-17	21%	12%	2,316,632,301	1,829,871,306
Feb-17	45%	24%	2,184,811,712	1,204,205,686
Mar-17	64%	20%	2,402,514,817	852,983,422
Apr-17	79%	14%	2,386,041,031	506,014,392
May-17	91%	12%	2,475,338,965	228,361,777
Jun-17	97%	6%	2,359,196,539	81,803,934
Jul-17	98%	1%	2,461,000,857	53,453,290
Aug-17	98%	0%	2,412,267,420	43,336,437
Sep-17	99%	0%	2,267,864,526	29,804,540
Oct-17	99%	0%	2,303,639,526	27,803,694
Nov-17	99%	0%	2,231,013,199	25,718,138
RYO			total thousands of kg sold	non-compliant* (thousands of kg sold)
Aug-16	1%		552287	546963
Sep-16	1%	0%	521917	516721
Oct-16	11%	10%	528560	469493
Nov-16	18%	7%	518188	424040
Dec-16	26%	8%	541359	400109
Jan-17	44%	18%	516174	288710
Feb-17	55%	11%	490464	218940
Mar-17	75%	20%	539695	132601
Apr-17	91%	16%	534343	45509
May-17	97%	5%	557734	19294
Jun-17	98%	2%	539118	9037
Jul-17	99%	1%	559731	5908
Aug-17	99%	0%	560994	4609
Sep-17	99%	0%	536870	2984
Oct-17	99%	0%	542052	2802
Nov-17	100%	0%	530219	2511

*RYO estimated to be sold in standardised packaging if sold in 30g or larger not pricemarked pack and (a) 30g pack of same variant on the market or (b) if 30g pack never available, pack on the market at end of analysis period)

For peer review only

Table S3 Menthol and capsule innovation

	Present at start		New Variant		Total	
	N	%	N	%	N	%
Menthol*						
FM premium	9	28%	2	20%	11	26.2
FM midprice	8	25%	0	0%	8	19.0
FM value	8	25%	4	40%	12	28.6
FM subvalue	3	9%	3	30%	6	14.3
no price segment	4	13%	1	10%	5	11.9
	32	76.2%	10	23.8%	42	100.0
Capsule**						
FM premium	2	15%	0	0%	2	10.5
FM midprice	2	15%	0	0%	2	10.5
FM value	9	69%	3	50%	12	63.2
FM subvalue	0	0%	3	50%	3	15.8
Total	13	68.4%	6	31.6%	19	100.0

*Menthol: variant name includes 'menthol' or 'green'

**Capsule: variant name includes 'capsule', 'crushball', 'dual', 'duo', 'demand', 'click on' or 'burst'

Table S4 Euromonitor estimates of the growth of cigars and cigariillos

	value constant £ mln	value current £ mln	volume million units	
2015	291.8	289.9	390.4	
2016	282.5	282.5	382.8	
2017	324.5	332.6	451.6	estimate
2018	347.8	366.3	493.8	estimate

For peer review only

Original protocol of the study

This paper is one of four covered by the original protocol. We have extracted sections relevant to this paper below and provided notes on where the eventual paper diverged.

Extracts from original protocol

Intended output

Changes in the number, names and visual appearance of packs (Research Question 1 (see below)): led by KER. A first draft will be produced by December 2017 based on analysis of the pack data and made available to CRUK at this point. Additional analyses of Nielsen and HMRC data[1] and the commercial literature ((see below) will contribute to a final paper on this topic which will be produced in December 2018.

Research Question 1: Changes in the number, names and visual appearance of brands and brand variants.

Pack purchasing data

Photographs will be taken of each surface of each pack and entered into NVivo for visual analysis[2]. We will use this to examine changes in packaging colours, shapes, product descriptors and fonts over time. We will specifically aim to explore whether these changes might be aimed to reassure smokers that standardised packaging does not mean compromises in taste or quality. An analysis of relevant articles in the commercial literature will be used to triangulate our findings.

Nielsen & HMRC[1] data

Building on evidence from Australia that a number of new brand variants were released by the tobacco industry prior to the enactment of standardised packaging, we will use both HMRC[1] and Nielsen data to provide a descriptive analysis of changes in the names and numbers of brand variants on the market, identifying the price segments within which the new brand variants sit. We will specifically seek to examine whether more descriptive brand names are introduced given that the pack design will no longer be able to convey messages of flavour, for example. The Nielsen interim extract (see below) will be used for this analysis which will be supported with our commercial literature review. We will present our findings using descriptive statistics and graphs.

Data sources:

Pack purchasing

Australian evidence suggests there were changes in the packaging of cigarettes prior to standardised packaging that would not be recorded in Nielsen data which only records brand variant names, price and volumes. For these reasons we propose to complement our Nielsen data with bespoke collected data.

For peer review only - http://bmjopen.bmj.com/site/about/guidelines.xhtml

We plan to buy, on a monthly basis, a leading brand from each of the four FMC price segments (premium, mid price, economy and ultra low price) and three RYO price segments (premium, mid price and economy)[3], to record changes over time in visual appearance of the packaging (including the font used for brand names, brand descriptors, colours, pack shapes) prior to standardised packaging becoming compulsory. We will supplement this with screen shots of brand variants from my supermarket.com, first using the purchased products to determine if the online pictures are regularly updated[2].

Robustness

This data will be collected by our research team. Collection will take place on the same day each month and from the same store. Store visits will also be used to explore changes in sale of pipe tobacco and whether RYO tobacco is being sold as pipe tobacco and if so, such products will be purchased too.

Availability and coverage

We will collect data from January[4] 2016 until May 2017 (the month when full implementation starts).

Commercial literature

We will update two reviews of the commercial literature we have recently undertaken applying the same review methodology. Specifically, we will review the following sources, identifying and reviewing articles on pricing, price segmentation, new brand variants and impacts of plain packaging on, inter alia, volumes and consumer behaviour.

We will use this literature in four ways. First, [not relevant to this paper]. Second, to record any discussion of standardised packaging and its impacts. This will be important in alerting us to any new lines of inquiry that might be needed to defend against emergent tobacco industry arguments. Third, it will be used to triangulate our findings, including those on pack appearance and new brand variants because the commercial literature is often used to flag new products or changes to existing products. Finally, [not relevant to this paper].

The literature review will cover the period from January 2015 to December 2017 and will include the following sources[5]:

- Retail publications:
    - The Grocer
    - Retail News
    - The Wholesaler
    - Convenience Store
    - Asian Trader
- Market and financial analyst reports:
    - Euromonitor reports
    - Citigroup reports
    - Redburn reports
- Tobacco company reports including:
    - Annual reports
    - Tobacco company presentations to analysts

Nielsen data[6]

For peer review only - http://bmjopen.bmj.com/site/about/guidelines.xhtml

As extracts cover three year periods we propose to only use data over a three year period in any particular analysis.

1) Interim extract

Dates: Sept 2014 to August 2017 (20 months pre-implementation period data, twelve months transition period, and three months full implementation period data).

This extract will be used for Research Question 1… Doing this on an interim extract will enable us to proceed more quickly with our analysis.

Notes on changes from protocol

1. HMRC data was only included as a possibility. We were eventually unable to obtain HMRC data.

2. The visual changes in the purchased packs were limited. Instead, we developed the novel advert analysis.

3. We included an extra BAT FM brand in order to include brands from all major tobacco companies rather than a brand from the small RYO value segment.

4. Funding was not confirmed until March 2016 when we started the purchasing.

5. Some sources were changed due to our understanding of work being undertaken by other research groups and due to insufficient focus on the UK market and/or standardised packaging in scoping work.

6. To understand product name changes we eventually compared product names in nine different Nielsen extracts in order to look for changes. At the time of analysis, the extract available to us allowed us to end our analyses in November 2017 giving us three extra months than was expected.

For peer review only - http://bmjopen.bmj.com/site/about/guidelines.xhtml

BMJ Open

A prospective longitudinal study of tobacco company adaptation to standardised packaging in the UK: identifying circumventions and closing loopholes

Journal:	*BMJ Open*
Manuscript ID	bmjopen-2018-028506.R1
Article Type:	Original research
Date Submitted by the Author:	05-Jun-2019
Complete List of Authors:	Evans-Reeves, Karen; University of Bath, Tobacco Control Research Group, Department for Health<br>Hiscock, Rosemary; University of Bath, Tobacco Control Research Group, Department for Health<br>Lauber, Kathrin; University of Bath, Tobacco Control Research Group, Department for Health<br>Gilmore, Anna; University of Bath, Tobacco Control Research Group, Department for Health
**Primary Subject Heading**:	Smoking and tobacco
Secondary Subject Heading:	Public health
Keywords:	PUBLIC HEALTH, PREVENTIVE MEDICINE, TOBACCO, STANDARDISED PACKAGING
Note: The following files were submitted by the author for peer review, but cannot be converted to PDF. You must view these files (e.g. movies) online.	
Video 1.MP4	

SCHOLARONE™
Manuscripts

A prospective longitudinal study of tobacco company adaptation to standardised packaging in the UK: identifying circumventions and closing loopholes

Karen A Evans-Reeves[1], email K.A.Evans-Reeves@bath.ac.uk

Rosemary Hiscock[1*], email R.Hiscock@bath.ac.uk

Kathrin Lauber[1], email K.Lauber@bath.ac.uk

Anna B Gilmore[1], email A.Gilmore@bath.ac.uk

[1]Tobacco Control Research Group, University of Bath and UK Centre of Tobacco and Alcohol Studies, Bath, UK

*Corresponding author: +44 (0)1225 388388

Key words: tobacco, tobacco industry, standardised packaging, plain packs

For peer review only - http://bmjopen.bmj.com/site/about/guidelines.xhtml

Abstract

Objectives: UK standardised packaging legislation was introduced alongside pack size and product descriptor restrictions of the European Union Tobacco Products Directive to end tobacco marketing and misinformation via the pack. This paper aims to assess compliance with the restrictions and identify attempts to continue to market tobacco products and perpetuate misperceptions of harm post-legislation.

Design, setting and intervention: A prospective study of the introduction of standardised packaging of tobacco products to the UK.

Participants and outcomes: We analysed commercial sales data to assess whether the legally required changes in pack branding, size and name were implemented. To explore any adaptations to products and packaging we analysed sales data, monthly pack purchases of factory-made cigarettes (FM) and roll-your-own tobacco (RYO) , tobacco advertisements from retail trade magazines and articles on tobacco from commercial literature (retail trade, market analyst and tobacco company publications).

Results: One month post full implementation of the UK and EU policies, 97% FM and 98% RYO was sold in compliant packaging. Nevertheless tobacco companies made adaptations to tobacco products which enabled continued brand differentiation after the legislation came into force. For example, flavour names previously associated with low tar were systematically changed to colour names arguably facilitating continued misperceptions about the relative harms of products. Tobacco companies used the one-year sell-through to their advantage by communicating brand name changes and providing financial incentives for retailers to buy large volumes of branded packs. In addition, tobacco companies continued to market their products to retailers and customers by innovating exemptions to the

For peer review only

legislation, namely, filters, packaging edges, seals, multipack outers, RYO accessories, cigars and pipe tobacco.

Conclusions: Tobacco companies adapted to packaging restrictions by innovating their tobacco products and marketing activities. These findings should enable policy makers globally to close loopholes and increase the potential efficacy of standardised packaging policies.

For peer review only

For peer review only - http://bmjopen.bmj.com/site/about/guidelines.xhtml

Article Summary

Strengths and limitations of the study

Strengths

- The strength of this paper lies both in the detail and depth of each analysis including our systematic analysis of retail-press advertisements (n=195) and commercial literature articles and reports (n= 396) alongside pack purchases of eight top-selling brands and detailed sales information from Nielsen.
- Using multiple data sources has enabled findings to be verified by more than one source and enabled a greater understanding of the tobacco industry's motives for changes and innovations to their products and packaging. This insight would not have been possible using one dataset in isolation.
- By using the commercial literature we were able to plug gaps in our knowledge, for example, we did not have Nielsen data on cigars or pipe tobacco but information in the retail and commercial literature revealed that these products were targets for innovation.

Limitations

- Nielsen data did not tell us whether RYO packs were in branded or standardised packaging. Given that a minimum of RYO pack size of 30g was a requirement of the TPD we assumed that new 30g RYO packs would switch to standardised packs at the same time.
- We do not know the extent to which Nielsen variant name changes lag behind and even reflect those printed on packs in retailers. However, we are reassured that the

For peer review only - http://bmjopen.bmj.com/site/about/guidelines.xhtml

main name change patterns found in the Nielsen data were similar to those found in a previous UK convenience store study.

Funding: This work was supported by Cancer Research UK grant number C27260/A23168. The authors are members of the UK Centre for Tobacco & Alcohol Studies, a UK Clinical Research Collaboration Public Health Research Centre of Excellence whose work is supported by funding from the Medical Research Council, British Heart Foundation, Cancer Research UK, Economic and Social Research Council, and the National Institute for Health Research under the auspices of the UK Clinical Research Collaboration (MR/K023195/1).

Competing Interests: The authors have no competing interests.

For peer review only

Background

Standardised tobacco packaging, one of the most significant policy threats to the tobacco industry in recent years, came into force in the UK on May 20th 2016. From this date all new factory made (FM) cigarettes and roll your own (RYO) tobacco, manufactured or imported for UK consumption were to be sold in standardised packaging. Tobacco companies were given until May 20th 2017 to comply with the law; a period referred to as the 'sell-through'. During this time, companies were not permitted to print any new branded packets(1, 2). The UK legislation(1) was implemented in concert with the revision of the 2001 European Union (EU) Tobacco Products Directive (TPD)(3) which placed further restrictions on packaging and naming of brands (Box 1)(3).

After Australia became the first country to introduce standardised packaging in December 2012, tobacco companies responded with more evocative and descriptive tobacco product names, including colours to represent the previous pack colour, thereby continuing the connotations associated with these colours(4, 5). Simultaneously companies reduced the total number of brands sold and renewed their focus on value for money brands by increasing the number and length of cigarettes in a pack and introducing menthol variants in this price range.(6) In response to standardised packaging in New Zealand and other markets there has been a significant increase in flavour capsule variants (FCVs) across all market price points,(7) which may threaten standardised packaging's effect on deterring smoking initiation given that these products appeal more to non-smokers and non-daily smokers than to daily smokers.(8, 9)

While the UK and EU legislation combined closed some of loopholes in the Australian legislation, by prohibiting product names that create an erroneous impression about the

For peer review only - http://bmjopen.bmj.com/site/about/guidelines.xhtml

health effects and requiring minimum pack sizes for both cigarettes and RYO,(10) concerns remain. The current legislation permits the use of colour descriptors, bevelled edges on packs, cigarette filter technology innovations and the TPD ban on menthol flavouring (including capsule technology) does not come into force until May 2020. Furthermore, cigars, cigarillos and pipe tobacco and RYO filter tips are exempt from the legislation and are still sold in branded packaging, while wholesaler multipacks are still allowed branded 'outer' wraps.

Recently published research suggests that, prior to the full implementation of standardised packaging in the UK, tobacco companies added smaller pack sizes such as 17 sticks to their brand variant portfolios,(11) introduced colour descriptors to brand family and brand variant names and made product changes, particularly to FM cigarette filters.(12) Examples of name change patterns associated with UK standardised packaging restrictions have been described qualitatively (10, 12, 13) but not quantitatively. The scale of these name changes and if/how these changes were communicated to the public is not clear from published research. Previous work has also only explored up to two months post-legislation.

Therefore, the purpose of this paper was to use mixed methods to combine data from four different data sources up to seven months post full-implementation to systematically and rigorously examine how compliant tobacco companies were with the legislation, and to explore any attempts they made to circumvent including any actions to reduce the immediate impact of the legislation during the sell-through. We assessed how the sell-through period was used, how and if tobacco company marketing adapted including any changes to products or packaging that would endure after the legislation was fully implemented, and whether and how products were marketed to retailers and consumers. The findings of this

For peer review only

paper have global significance as they can help close loopholes during the design and implementation of standardised packaging legislation in other jurisdictions.

For peer review only

For peer review only - http://bmjopen.bmj.com/site/about/guidelines.xhtml

BOX 1. Summary of restrictions UK Standardised packaging legislation and the European Union Tobacco Products Directive Revision (implemented 20 May 2016 – 20 May 2017)

EU TOBACCO PRODUCTS DIRECTIVE
PACKAGING OPENING: A unit packet of cigarettes may consist of carton or soft material and shall not have an opening that can be reclosed or re-sealed after it is first opened, other than the flip-top lid and shoulder box with a hinged lid. For packets with a flip-top lid and hinged lid, the lid shall be hinged only at the back of the unit packet.
PACK SIZE: Cigarette packs must contain a minimum of 20 cigarettes & RYO tobacco must contain at least 30 grams of tobacco.
IMPRESSION CREATION: There must be no packaging elements that create an erroneous impression about the characteristics, health effects, risks or emissions of tobacco.
TOBACCO INGREDIENTS: No description of nicotine, tar or carbon monoxide content of a tobacco product.
FLAVOUR DESCRIPTION: No reference to taste, smell or any flavourings or additives or the absence of any such thing.<br>FLAVOURINGS (May 2020): No flavourings in any components such as filters, papers, packages, capsules or any technical features allowing modification of the smell or taste of tobacco products.
ENVIRONMENT: No suggestion that a particular tobacco product has improved biodegradability or other environmental advantages.
RESEMBLANCE: No resemblance to a food or cosmetic product.
PROMOTION: No suggestion of economic advantage of one product over another – no price marked packs.
UK STANDARDARDISED PACKAGING
PACK EXTERNAL COLOUR: External packaging of cigarettes or RYO tobacco is Pantone 448C (matt finish).
PACK INTERNAL COLOUR: Internal colour of cigarette or RYO packaging must be white or Pantone 448C (matt finish).

For peer review only - http://bmjopen.bmj.com/site/about/guidelines.xhtml

NOISE AND SMELL: Packaging must not make a noise or produce a smell that is not normally associated with the packaging.
PACKAGING EVOLUTION: The packaging must not change after retail sale, for example, heat activated inks, embellishments designed to appear gradually over time, scratch panels etc.
PACKAGING SHAPE: Cigarette packets must be made of carton or soft material and be cuboid in shape & RYO may be in a cuboid box, a cylinder or a pouch (bevelled or rounded edges are permitted).
STICK DESIGN: Cigarette stick paper must be plain white (matt finish), the filter must only be coloured in such a way as to imitate cork. The stick may have the brand name and variant printed in black Helvetica type no larger than 8 point in normal weighted regular typeface not more than 38 mm from the filter end of the cigarette. The start of each word can be an upper case letter but the rest of the word must be lower-case.

For peer review only - http://bmjopen.bmj.com/site/about/guidelines.xhtml

Methods

Data Sources

We utilised four data sources, 1) purchases of eight top-selling tobacco brands; 2) tobacco advertisements in popular retail trade publications; 3) other commercial literature: retail press articles and advertorials, tobacco company annual reports and Euromonitor market analyst reports; and 4) Nielsen data.(Figure 1) While compliance with the legislation was primarily assessed using the Nielsen data, the evidence collected from each data source was utilised to assess whether, and how, tobacco companies adapted to the legislation and continued to market their products. We triangulated data from these different datasets, to verify the existence and purpose of adaptations.

Pack Purchasing and observation

We conducted monthly pack purchases between March 2016 and May 2017 to assess visual and sensory changes over time in the top-selling FM and RYO products (Table 1) for different price segments defined from commercial literature and Nielsen data tobacco price data of sales between 2008 and early 2016(11). We purchased packs from five different FM price segments and two different RYO price segments. We also included BAT's top selling brand in the UK as an addition as BAT did not have a top-selling brand in any of the identified price-segments.

Additionally, we observed price lists of cigar and pipe tobacco brands available in-store and the appearance of 'outers' (the cover wrap used to bind multiple packs of RYO or FM together for distribution) where visible.

For peer review only - http://bmjopen.bmj.com/site/about/guidelines.xhtml

Table 1. Pack purchase data of the top selling brand in each price segment

Price segment (average price per pack/stick) [1] [ref Nielsen paper]	Top selling products name & packsize		Co [4]
	March 2016 (start of sell-through)	May 2017 (full-implementation)	
FM			
Premium (£6.76/£0.42)	Marlboro Gold (20s)	Marlboro Gold (20s)	PMI
Midprice [2] (£5.61/£0.36)	L&B Original Lambert & Butler Silver (20s)<br>Mayfair King Size (19s)	Lambert & Butler Original Silver (20s)<br>Mayfair King Size (20s)	IMT<br>JTI
Value (£5.10/£0.33)	L&B Blue Lambert & Butler Real Blue (19s)	L&B Blue Real Blue(20s)	IMT
Subvalue (£4.68/£0.29)	Carlton Red (19s)<br>Rothmans of London[3] (17s)	Carlton Red (20s)<br>Rothmans Blue (20s)	IMT<br>BAT
RYO[5]			
Premium (£6.97/£0.17)	Golden Virginia The Original (25g)	Golden Virginia The Original (30g)	IMT
Midprice (£5.81/£0.15)	Amber Leaf (25g)	Amber Leaf Original (30g)	JTI

[1] weighted average price per pack/stick of all products sold within tobacco industry price segments as described by previous analysis (Dec 2015 prices – inflation adjusted to 2008 values). One RYO stick was estimated to be 0.5g tobacco(11, 14).

For peer review only - http://bmjopen.bmj.com/site/about/guidelines.xhtml

[2]Midprice was originally two major segments: Lambert & Butler was the leading upper midprice product and Mayfair the leading lower midprice product

[3] None of the top selling brands in each price range was owned by British American Tobacco. For completeness we added Rothman's of London (a subvalue brand) - the top selling brand for British American Tobacco.

[4]Tobacco company' abbreviations: BAT British American Tobacco, IMT Imperial Tobacco, JTI Japan Tobacco International, PMI Philip Morris International

[5] No RYO value segment (average pack/stick price: £4.28/£0.14) product was purchased due to lower sales volumes even of the leading brand (Gold Leaf) in this segment.

Advertisements in the retail press

We searched hard copies of the most widely circulated paid for retail and wholesale publications *The Grocer, Wholesale News* and *Retail Newsagent* between January 2015 and December 2017 for all tobacco advertisements.

We coded 195 advertisements for the attributes marketed to retailers, e.g., new brand variant, packaging, filter, limited edition, price, retailer profit. We examined the distribution of these marketing themes by price segment (Table S1). We separately assessed advertorials for relevance to standardised packaging and noted the main messages communicated.

Commercial literature review

We searched high circulation online retail publications (*The Grocer, Wholesale News, betterretailing.com* (which includes *Retail Newsagent* and *Retail Express, talkingretail.com*), to identify articles relevant to tobacco packaging and marketing (Jan 2015 – Dec 2017; Table 2). We also included two reviews of 2017 published in 2018, Euromonitor reports on tobacco, and tobacco company annual reports covering this period. We used NVivo 10, to

For peer review only - http://bmjopen.bmj.com/site/about/guidelines.xhtml

code articles for any evidence of circumvention of the legislation and used quotes taken from the commercial literature as examples of each circumvention (Table S2).

For peer review only

For peer review only - http://bmjopen.bmj.com/site/about/guidelines.xhtml

Table 2 Commercial literature review January 2015 to December 2017

Source	*Search terms (depends on search procedure of website)*	*Articles/reports found*	*Articles/reports included in final analysis*
**RETAILER MAGAZINES**			
**Convenience & independents**			
Betterretailing.com: Retail Newsagent Retail Express	"tobacco"	350	181
TalkingRetail.com	"tobacco"	50	49
**Wholesalers &FMCG**			
Wholesale News	"tobacco" or "cigarette" or "ryo" or "roll+your+own" or "hand+rolled" or "cigar" or "cigarillo"	54	30
The Grocer	"tobacco" or "cigarette" or "ryo" or "roll+your+own" or "hand+rolled" or "cigar" or "cigarillo"	820	104
**INDUSTRY ANALYST**			
Euromonitor Passport	"tobacco"	NA	20
**TOBACCO COMPANY**			
Annual reports from the big four tobacco companies: JTI, IMT, BAT and PMI	Annual reports	12	12
**TOTAL**		1286	396

Nielsen Data

For peer review only - http://bmjopen.bmj.com/site/about/guidelines.xhtml

For peer review only

Nielsen collates data on tobacco sales from nearly 90% of UK supermarkets and a stratified sample of 15% of convenience stores(11). For each product, Nielsen records hierarchically the tobacco brand, brand family, brand variant and then specific features of the pack denoted by a unique serial number known as a SKU (e.g. size, pricemarked)(11). In November 2017 there were 71 brands, 97 families, 241 variants, and 1022 SKUs (Table 3).

For peer review only

For peer review only - http://bmjopen.bmj.com/site/about/guidelines.xhtml

Table 3 Hierarchical brand architecture available from the Nielsen data

		N (UK market)	
**Hierarchy of Nielsen data**	**Example**	**July 2014**	**Nov 2017**
BRAND	Marlboro	44	71
BRAND FAMILY Products with the same brand name but sold at different price points	Marlboro Bright Leaf	114	97
BRAND VARIANT Product at same price point but with different length (e.g. superkings, kingsize), flavour or other characteristics	Marlboro Bright Leaf Platinum	282	241
Stock Keeping Unit (SKU) Each individual barcoded product including specific pack characteristics: pack size, whether the pack is price-marked, and, for FM but not RYO, whether standardised or branded	Marlboro Bright Leaf Platinum 10s multipack not price marked	930	1022

Data Analysis

Compliance

For peer review only - http://bmjopen.bmj.com/site/about/guidelines.xhtml

Monthly Nielsen sales data were used to examine compliance with the removal of branding, pricemarking and small pack sizes (Box 1), between July 2016 (when the first standardised packs first were sold)(13) and November 2017. For FM, Nielsen data identifies which packs are standardised and which branded. For RYO they do not. However, as 30g packs of RYO were negligible prior to standardised pack legislation RYO packs of 30g or more were treated as standardised.

Name changes

We identified the number, names and price segments of all brand variants whose name changed between July 2014 and November 2017. July 2014 was used as a starting point for this analysis as we wanted a time before standardised packaging was passed by the Government in January 2015. We coded and counted whether each name change was required for compliance with the legislation and recorded the type of name change eg colour added or adjective added.

For peer review only

For peer review only - http://bmjopen.bmj.com/site/about/guidelines.xhtml

Innovation targeting

To understand whether innovations were targeted at particular price segments, we compared the distribution of name changes and new variants in November 2017 with the distribution of price segments prior to the decision to implement legislation in July 2014. Differences in the number of variants were tested using chi-square tests and Fishers exact tests (when the expected number of cases was less than five). To achieve sufficient numbers for analysis, price segments were merged into four categories: (1) FM premium and midprice, (2) FM value and subvalue, (3) RYO premium and midprice, (4) RYO value.

Patient and Public Involvement

KER and RH discussed standardised packaging policy with the UK Centre for Tobacco and Alcohol Studies University of Nottingham panel of smokers and former smokers (now known as the Tobacco & Nicotine Discussion Group) in October 2017. The group were asked to discuss their thoughts on and experiences of the policy and whether they had noticed any changes to tobacco products and packs both in the sell-off period and thereafter. These discussions helped inform our research questions but we did not include these discussions in our analysis. Our study did not involve human subjects and therefore we did not seek any further involvement from the panel.

Results

Four main strategies were identified from multiple data sources. We present the evidence for each in turn.

Strategy 1: Keep branded packets on the market as long as possible

For peer review only - http://bmjopen.bmj.com/site/about/guidelines.xhtml

Six months into the year-long sell-through period, 96% of FM and 82% RYO were still sold in branded packs (Figure 2 & table S3). Most FM (70%) and RYO (65%) packs switched to standardised packs between January and April 2017. One month after the full-implementation of the UK and EU policies, 97% of cigarettes and 98% of RYO were sold in compliant packaging. By November 2017, non-compliant sales declined to 1% of FM and negligible volumes of RYO.

The commercial literature suggests that tobacco companies produced greater volumes of their branded FM and RYO tobacco prior to the May 2016 manufacturing deadline to keep branded packaging on the market for as long as possible during the sell-through(15). Both the Nielsen data and the adverts and the commercial literature revealed an increase in small pack sizes ahead of the sell-through period. Tobacco companies offered incentives and promotions on branded packs to retailers to encourage sales and loyalty to their products, examples included cash rewards and loyalty points for selling particular brands. By early 2017 manufacturers were described as being in a price-war(16, 17). To allay retailers' concerns about being left with non-compliant stock after the deadline, tobacco companies offered to buy them back (Table S2; Strategy 1.3)(18).

Overall, tobacco companies decreased the number of brand families and brand variants between July 2014 and November 2017 as they were consolidating their portfolios and focusing on the brands which could offer growth. The increase in SKU between 2014 and 2017 reflects (a) the pre-standardised packs increase in small pack sizes, innovations such as flavours and capsules (b) the addition of standardised packs FM SKU and 30g RYO SKU

For peer review only - http://bmjopen.bmj.com/site/about/guidelines.xhtml

For peer review only

and (c) that many SKU in branded packs remained on the market in November 2017 but selling only tiny volumes.

Strategy 2: Maintaining brand variant differentiation through name changes

One third (35%; 123/ 353) of variants changed name between July 2014 and November 2017. Less than half of these changed name to comply with legislation (42%, n=52/123) and most of these removed flavour names as the legislation required but substituted with other names, most often a colour (Table 4). Most notably, FM smooth became bright or sky blue, with RYO smooth changing to yellow and menthol for both FM and RYO changing to green.

Of the variants which changed name when it was not necessary for legislative compliance (N=71/123, 58%), colours were introduced for the first time as a substitute for the previous pack colour, eg. *Marlboro* with the red chevron on the pack became Marlboro Red. Similarly, to distinguish brand variants from one another and to continue previous brand variant identities, other adjectives were added to brand names, most notably adjectives accompanied a colour, e.g. *real* blue, *bright* silver, *legendary* black. In addition, during the study period, 23 new variants appeared in the Nielsen dataset with names which included elements that were novel: 'signature', 'ome', 'silver strand' in May 2015, 'Eagle,' and 'Colours'' in November 2015 and 'Chill' in May 2016 and two different brands added 'Black Russian' variants in November 2015 and January 2017. Lastly, two new Golden Virginia RYO variants appeared on the market in November 2015 named 'Midnight' and 'Sunrise'. Commercial literature revealed that these indicated rich and smooth flavours respectively(19). The first brand variant in a brand family sometimes had 'original' added e.g. Amber Leaf RYO became Amber Leaf original.

For peer review only - http://bmjopen.bmj.com/site/about/guidelines.xhtml

Table 4. Nielsen data FM and RYO brand variant name changes from (July 2014 - November 2017)

		N	Additions/substitutions
**Legally required actions**			
Taste removed	*Replacement*		
Menthol	Colour	20	green
	No replacement	2	
Smooth	Colour + adjective	12	bright blue/yellow, sky blue
	Adjective	5	bright, sky
	Colour	3	blue, yellow
	Other Substitution	1	fine
other taste (‘fresh burst’, ‘fresh taste’, ‘ice capsule’, ‘subtle flavour‘)	Other Substitution	3	crushball, dual
	No replacement	1	
Environmental advantages removed			
‘Natural’	Other Substitution	2	blue, king size
	No replacement	3	
Total		52	
**Discretionary actions only**			
colour+adjective added		9	original silver/blue/black, real blue/red
adjective added		8	bright, real, legendary
colour added		7	blue, red, black
‘original’ added		6	

For peer review only - http://bmjopen.bmj.com/site/about/guidelines.xhtml

size (king size, superkings) removed	6	
Mixed & miscellaneous	35	Mixed: e.g. colour added and size removed<br>Miscellaneous: e.g. location added or removed e.g Rothman's of London became Rothman's Blue
Total	71	
**Grand total**	**12 3**	

In October 2016, five months into the sell-through period and before IMT had made any of its legally required name changes to its products, the company outlined its brand renaming strategy in an advertorial in the retail press(20). The advertorial explained that all products traditionally presented as full flavour would become 'real' or 'original' and be associated primarily with the colour red. Those previously denoted as 'smooth' would change to 'bright' and become associated with the colour blue. Menthol would become green and capsule would become Crushball (Table S2: Strategy 2). IMT also provided retailers with materials to help explain changes to consumers(20). Some companies also advertised name changes directly to consumers through pack inserts (eg. BAT's Rothmans of London included a pack insert to warn its smokers that it was to become Rothman's Blue).

Strategy 3. Focus on lower price segments

During the study period, 68% of new variants recorded by Nielsen were FM and 32% RYO. Given that in July 2014, 81% variants on the UK market were FM and 19% were RYO (Table 5), more new RYO variants were introduced than expected (p=.018).

For peer review only - http://bmjopen.bmj.com/site/about/guidelines.xhtml

Half (50%) of all new variants were introduced in the FM value and subvalue price segments, 22% FM premium and midprice, 15% RYO premium and midprice and 13% RYO value. However, prior to the decision to implement standardised packaging (July 2014), the distribution of the market was 50% FM premium and midprice, 35% FM value and subvalue, 8% RYO premium and midprice and 7% RYO value. Thus new variants were under-represented among FM premium and midprice brands (p=.001).

Similarly, compared with July 2014, name changes in the study period were overrepresented among FM value and subvalue (p=.048) and under-represented among FM premium and midprice brands (p=.002).

Companies introduced more menthol and flavoured capsule variants to the lower price segments which traditionally offered fewer of these products compared to premium and midprice segments. Since July 2014, 13 menthol or flavoured capsule variants were introduced to the value and subvalue price segments compared to just two new brand variants in the premium and midprice range (Table S4). Overall, the number of brand families and brand variants declined. Tobacco companies reported concentrating on fewer brand families so that they could improve the “quality of growth” and “cut the level of complexity and cost in the business.”(21)

For peer review only

For peer review only - http://bmjopen.bmj.com/site/about/guidelines.xhtml

Table 5 Nielsen data. Price segmentation of new variants and variant names changes between July 2014 and November 2017 compared to July 2014 baseline.

	Baseline distribution of variants (July 2014)		Name changes (July 2014 – November 2017)			New variants (July 2014 – November 2017)		
	N	%	N	%	P*	N	%	P*
**Tobacco Type**								
FM			12		.527			.018
	225	81%	5	79%		48	68%	
RYO	54	19%	35	21%		23	32%	
**Price segment**								
FM premium & midprice	103	50%	48	33%	.002	10	22%	.001
FM value & sub value	72	35%	66	45%	.048	23	50%	.054
RYO premium & midprice					.097			.164
	17	8%	20	14%		7	15%	#
RYO value					.735			.602
	15	7%	12	8%		6	13%	#

*Chi square comparing with July 2014 distribution

#Fishers exact test comparing with July 2014 distribution

Nearly two-thirds of the advertisements captured from the retail press, 124/195 (64%) advertised value or subvalue brands. Similarly, for new products, the majority of advertisements (30/38; 79%) were for value or subvalue brands. Only one advert was a new premium offering.

The commercial literature found tobacco companies innovating within lower price segments, introducing packs with fewer cigarettes prior to the sell-through (22-24) followed by a price-war in the lower priced segments with some manufacturers reportedly selling at a loss to gain market share(16). By the end of 2017 tobacco companies stated that smokers expected better quality products in lower price segments after standardised packaging and Minimum Excise Tax (MET) legislation raised the price of the cheaper brands and so ‘premium

For peer review only

features' in lower priced cigarettes appeared (Table S2: Strategy 3)(23, 25). For example, a redesign to Chesterfield (subvalue) was advertised to retailers in December 2017, citing a new 'firm' filter and, as with premium brand Marlboro, a new bevelled edge box(24, 26).

For peer review only

For peer review only - http://bmjopen.bmj.com/site/about/guidelines.xhtml

Strategy 4. Innovating exemptions to the legislation

The commercial literature and pack purchase study suggested that tobacco companies continued to innovate and market their tobacco products by focusing on exemptions to the legislation (Table S2: Strategy 4).

Pack modifications that endured post-legislation

During the sell-through, the pack purchase and commercial literature review revealed that FM Marlboro 10s and RYO *Amber Leaf*, *Golden Virginia* and *Cutter's Choice* were sold in reusable tins, arguably allowing consumers to decant cigarettes bought post-legislation into branded tins. Selling RYO in tins is not novel but cigarettes have rarely been sold in tins previously.

Pack purchases found that best-selling premium brand family *Marlboro* changed significantly in July 2016 (two months into sell-through), with the introduction of bevelled pack edges and a *pro-seal* closing mechanism (Figure 3). Both features fundamentally changed the tactile nature of the pack which endured after full-implementation in May 2017 (Supplementary Video 1). To be compliant with the legislation, these packs must have been printed prior to May 2016.

Extra Sticks

Given that there is no restriction on the maximum number of sticks per pack, packs with more than 20 continued post-legislation. During the sell-through (May 2016 - May 2017) sales of packs with 23 and 24 sticks increased from 7 to 18 million sticks. Sales peaked at 25 million sticks in August 2017 and declined to 21 million sticks in November 2017.

For peer review only - http://bmjopen.bmj.com/site/about/guidelines.xhtml

Branded Outers

The retail literature and in person observations revealed that multipack wholesale outers for FM and RYO are branded (Figure 4). Outers can be seen by customers during tobacco product gantry restocking. Advertisements in the retail press showed images of branded tobacco products including branded outers post-legislation.

RYO accessories

Increased innovation was observed among RYO accessories post legislation with retail press articles in November 2017 and February 2018 referring to "'ultra slim' and slim filters and papers, biodegradable filters, and menthol tips" (Table S2; Strategy 4.4) (25, 27).

Cigars

Cigars, which can be sold in branded packs, single sticks without pictorial health warnings, at a relatively low price and with good profit margin, were identified as an opportunity for growth (19, 24, 28-30). Euromonitor's models suggested growth in cigars and cigarillos sales volumes (390 to 494 million units) and value (£292 to £348 million) from 2015 to 18 (Table S5). Towards the end of 2017, JTI and cigar company Ritmeester were holding social events to build relationships with retailers (Table S2; Strategy 4.5) (24).

Pipe Tobacco

The retail literature revealed that one small tobacco manufacturer, *Gawith Hoggarth*, deliberately marketed pipe tobacco as RYO to circumvent the minimum pack size restrictions on RYO (Table S2; Strategy 4.6)(31).

Discussion

For peer review only - http://bmjopen.bmj.com/site/about/guidelines.xhtml

UK Standardised Packaging legislation and the EU TPD placed restrictions on tobacco packaging and marketing in the UK. Overall, although compliance with the restrictions (removal of branding, small pack sizes and non-compliant names) was not 100% one month after full implementation, the majority of non-compliance could be explained by less popular tobacco products that were not widely circulated. However, clarity is needed about the legality of isolated incidents such as the compliance of the *Marlboro* resealable pack and Gawith Hoggath's marketing of its *Kendal* pipe tobacco as RYO (Video 1).

Technical compliance aside, this paper identified four key strategies use by tobacco companies to circumvent the legislation.

Firstly, the retail literature suggests that tobacco companies used the 12-month sell-through to their advantage, keeping branded stock on the market as long as possible, and using the time to communicate name changes and new brand variants to retailers and customers. Ahead of the sell-through companies increased production of branded packs, introduced smaller pack sizes to enable more affordable offerings, and encouraged retailers to buy large quantities of branded stock at reduced prices. Other countries had a shorter sell-through period with three-months for Australia and New Zealand (32, 33) and seven months for France (34). Governments considering the policy in other countries should therefore consider mandating a short sell-through period.

Second, although compliant with legally required name removals, tobacco companies implemented a standard name change formula that maintained the brand identity and differentiation of the three broad flavour categories (1) full flavour; (2) smooth; (3) menthol(20). In 2002 terms such as 'mild' and 'light' were prohibited to curb misperceptions of relative harm. However, the continuation of the colours of the previous packs such as the

For peer review only - http://bmjopen.bmj.com/site/about/guidelines.xhtml

gold and white pack for *Marlboro 'lights'* and the introduction of terms such as smooth for other brands sustained these misperceptions(35, 36). In line with the power of colour in brand identity,(36-38) this study shows that tobacco companies changed '*full-flavour'* variants to *red, original,* or *real; menthol* variants to *green*; and *smooth variants* to *bright* and *blue (*or *yellow* for RYO). As in 2002, the current restrictions on tobacco product name were designed disrupt misperceptions of relative harms based on flavour descriptions. However, by maintaining the broad flavour categories such misperceptions are likely to endure. Additionally, cigarette packs with filter descriptions such as 'advanced' and 'firm' filter are perceived as less harmful by existing smokers and therefore perpetuate the perceptions that some tobacco brand variants are less harmful than others(39). Given that companies continue to innovate their product names and descriptions it may be considered necessary to follow the example of Uruguay where only one brand variant is permitted per brand and no new brands are allowed(40).

Third, Nielsen data and the commercial literature revealed that tobacco companies were fighting fervently for market share in the cheaper price segments with a price war pre implementation. In the lower price segments, tobacco companies implemented more name changes, introduced more new brand variants(including menthol and flavour capsules) and more RYO variants and placed more advertisements for lower priced products then they did for premium and midprice products. Other work suggests that RYO is a lower cost alternative to smokers who may otherwise quit and so this may explain the introduction of more RYO offerings in lower price segments (41-43). Similarly, menthol and particularly flavour-capsule variants appeal more to non-smokers and non-daily smokers compared to smokers and may therefore recruit non-smokers and increase overall smoking prevalence (8). Flavoured cigarettes including capsule flavourings will be outlawed in the EU by May

For peer review only - http://bmjopen.bmj.com/site/about/guidelines.xhtml

2020. With the UK set to leave the EU in 2019, the tobacco industry may utilise this opportunity to try and roll back tobacco control regulations made under the EU TPD.

Our fourth finding that tobacco companies are innovating tobacco products, features and accessories not covered by the UK or EU legislation for growth provides may provide a rationale for expanding the legislation to include products such as RYO filters and papers, standardised packaging for cigars, cigarillos, pipe tobacco, wholesale outer wraps, standardised pack edges, maximum pack sizes and prohibitions for innovations to pack seals. Tobacco companies, such as Imperial Tobacco in the UK have recently introduced a whole series of flavour capsule variants to their *Rizla* filter tip product offerings for RYO tobacco.

The strength of this paper lies both in the detail and depth of each analysis including our systematic analysis of retail press advertisements (n=195) and commercial literature articles and reports (n= 396) alongside pack purchases of the top selling brands and detailed sales information from Nielsen. Using multiple data sources enabled findings to be verified by more than one source and enabled a greater understanding of the tobacco industry's motives for any changes made to their products and packaging. In addition to the evidence presented by others [1, 13, 14], by following these data sources up to seven months post-legislation we were able to observe tobacco companies increased focus on innovations to exemptions to the legislation that offered opportunities for growth e.g. RYO filters and accessories and cigars.

Nevertheless, despite evidence from an IMT whistleblower (44) and PMI's own words (45), our data did not reveal price mark stickers on tobacco products. This is likely to be because we bought our eight brands from a large supermarket and not a convenience store and

For peer review only - http://bmjopen.bmj.com/site/about/guidelines.xhtml

because this industry strategy was not highlighted in the retail literature due to the questionable legality of this strategy(46).

Resources prevented us from acquiring Nielsen data on cigars or pipe tobacco and from purchasing more than one brand per price segment in the pack purchasing study element. However, the retail literature alerted us that cigars and other products were targeted as growth opportunities and, although not able to fully capture the sensory nature of brands, our analysis of the advertisements ensured that we saw many, if not all, innovations being promoted to retailers.

Nielsen data do not record whether RYO products are sold in standardised packs. We assumed that 30g packs were always in standardised packs and that larger packs would switch to standardised packs at the same time. It is possible that this did not occur. However the temporal patterns of name changes and pack sizes in the Nielsen data were similar for FM and RYO and it is therefore reasonable to assume that branding was removed at the same time. Nielsen model prices and volumes for the UK based on a census of the major supermarket sales and a rolling sample of convenience stores. Although, we do not know the extent to which Nielsen variant name changes lag behind and even reflect those printed on packs in retailers, the main name change patterns found in the Nielsen data were similar to those found in a UK convenience store study(47) and two evaluations of the introduction of standardised packaging in Australia(4, 5).

Given that the tobacco industry is attempting to circumvent standardised packaging legislation, other countries considering the policy should consider how to make regulations as comprehensive as possible to prevent the exploitation of continued marketing opportunities. In summary, the evidence in this paper suggests a number of possible policy

For peer review only - http://bmjopen.bmj.com/site/about/guidelines.xhtml

options, namely that long sell-through periods should be avoided and that restricting tobacco products to one brand variant per brand may be the only comprehensive way to prevent misperceptions of harm and an that extending regulations to include other tobacco products, features and accessories should be considered.

For peer review only

For peer review only - http://bmjopen.bmj.com/site/about/guidelines.xhtml

Author contributions: AG designed the study with contributions from KER and RH. KER led the writing of the paper and designed, conducted and analysed the pack purchase element of the study. RH analysed the Nielsen data in addition to downloading and analysing the commercial literature. KL collated the retail literature advertisements, involving visits to the British Library and analysed the data. All authors read and approved the manuscript.

Acknowledgements: The authors would like to thank Dr Rob Branston who read and commented upon a near final draft and Ilhan Marsal who helped with early preparation of the advertisements and pack purchased data. We would also like to acknowledge Nielsen (UK) and Public Health England for provision of the sales data.

Data statement:

1. The Nielsen dataset: cannot be accessed by others as it is paid for data and we do not have permissions to republish these.
2. Pack purchasing: qualitative observational data are available from the University of Bath data archive
3. Retail press advertisements: the coded dataset is available from the University of Bath data archive
4. Commercial literature: full articles were analysed in NVivo software. As the majority of documents came from paid for publications we are not permitted to publish these articles. Tobacco company annual reports are publicly available on tobacco company websites.

For peer review only

For peer review only

For peer review only - http://bmjopen.bmj.com/site/about/guidelines.xhtml

References

1. The standardised packaging of tobacco products regulations 2015, No.829, (19 March 2015). Available from: https://www.legislation.gov.uk/uksi/2015/829/pdfs/uksi_20150829_en.pdf.
2. Department of Health. Tobacco Packaging Guidance: Guidance for retailers, manufacturers and distributors of tobacco products, enforcement agencies and the public on changes to tobacco packaging from 20 May 2016 2016 [updated May 2017, cited 8 January 2018]. Available from: https://www.gov.uk/government/uploads/system/uploads/attachment_data/file/596139/Tobacco_Packaging_Guidance.pdf.
3. Directive 2014/40/EU of the European Parliament and of the Council of 3 April 2014, (2014). Available from: http://eur-lex.europa.eu/legal-content/EN/TXT/PDF/?uri=OJ:JOL_2014_127_R_0001&from=EN.
4. Scollo M, Occleston J, Bayly M, et al. Tobacco product developments coinciding with the implementation of plain packaging in Australia. *Tobacco Control*. 2014;24:e116-e22.
5. Scollo M, Bayly M, White S, et al. Tobacco product developments in the Australian market in the 4 years following plain packaging. *Tobacco Control*. 2018;27:580-4.
6. Scollo M, Zacher M, Coomber K, et al. Changes in use of types of tobacco products by pack sizes and price segments, prices paid and consumption following the introduction of plain packaging in Australia. *Tobacco Control*. 2015;24(Suppl 2):ii66-ii75.
7. Haggart K, Hoek J, Blank M-L. Flavour capsule variants' performance in a "dark market": Implications for standardised packaging. *Nicotine & Tobacco Research*. 2018;158.
8. Hoek J, Gendall P, Eckert C, et al. Young adult susceptible non-smokers' and smokers' responses to capsule cigarettes. *Tobacco Control*. 2018;0:1-8.
9. Moodie C, Ford A, Dobbie F, et al. The Power of Product Innovation: Smokers' Perceptions of Capsule Cigarettes. *Nicotine & Tobacco Research*. 2017;20(9):1157-60.
10. Moodie C, Hoek J, Scheffels J, et al. Plain packaging: legislative differences in Australia, France, the UK, New Zealand and Norway, and options for strengthening regulations. *Tob Control*. 2018.
11. Hiscock R, Branston JR, McNeill A, et al. Tobacco industry strategies undermine government tax policy: evidence from commercial data. *Tobacco Control*. 2017;27:488-97.
12. Moodie C, Angus K, Mitchell D, et al. How tobacco comapnies in the UK prepared for and responded to standardised packaging of cigarettes and rolling tobacco. *Tobacco Control* 2018.
13. Breton MO, Britton J, Huang Y, et al. Cigarette brand diversity and price changes during the implementation of plain packaging in the United Kingdom. *Addiction*. 2018.
14. Branston JR, McNeill A, Gilmore AB, et al. Keeping smoking affordable in higher tax environments via smoking thinner roll-your-own cigarettes: Findings from the International Tobacco Control Four Country Survey 2006–15. *Drug and Alcohol Dependence*. 2018;193:110-16.
15. Explained: Retailer's questions about EUTPD II: Retail Newsagent; 10 May 2016 [18 April 2018]. Available from: https://www.betterretailing.com/questions-about-eutpd2.
16. Value brands price war hits retail profits: BetterRetailing; 17 February 2017 [18 April 2018]. Available from: https://www.betterretailing.com/value-brands-price-war-hits-retail-profits.
17. Cash incentives and price cuts drive tobacco sales: Retail Newsagent; 26 May 2016 [20 April 2018]. Available from: https://www.betterretailing.com/tobacco-cashandcuts.
18. Samrai G. Wholesalers: tobacco suppliers will help retailers with branded stock post-EUTPD II: Retail Newsagent; 2 March 2017 [18 April 2018]. Available from: https://www.betterretailing.com/tobacco-suppliers-branded-stock.
19. Dillon C. Three things you can learn from Ritmeester's cigar seminars: Retail Express; 17 September 2017 [24 April 2018]. Available from: https://www.betterretailing.com/ritmeesters-seminar.
20. Imperial Tobacco. Partnering for Success through EUTPD II and standardised packaging: Part 4. New pack descriptors under EUTPD II. Retail Newsagent. 28 October 2016:20-1.

For peer review only - http://bmjopen.bmj.com/site/about/guidelines.xhtml

21. Imperial Brands. Annual report and accounts 2017: Investing for growth 2017 [29 May 2019]. Available from: https://www.imperialbrandsplc.com/content/dam/imperial-brands/corporate/investors/annual-report-and-accounts/online-annual-report-pdfs/Annual_Report_2017_Final_PDF_Web_Ready_171207.pdf
22. O'Donnell T. The fat lady sings: tobacco category report 2016: The Grocer; 11 February 2016 [19 April 2018]. Available from: https://mweb02.thegrocer.co.uk/reports/category-reports/the-fat-lady-sings-tobacco-category-report-2016/531520.article.
23. Selwood D. Pall Mall cigarettes get taste plus filter in range upgrade 18 April 2016 [20 April 2018]. Available from: https://www.thegrocer.co.uk/buying-and-supplying/marketing/pall-mall-cigs-get-taste-plus-filter-in-range-upgrade/534748.article.
24. Yau A. The latest developments from the biggest tobacco brands: Retail Newsagent; 5 April 2018 [20 April 2018]. Available from: https://www.betterretailing.com/developments-from-the-biggest-tobacco-brands.
25. Pendleton R. A tightening stranglehold: tobacco category report 2018: The Grocer; 9 February 2018 [19 April 2018]. Available from: https://www.thegrocer.co.uk/buying-and-supplying/categories/tobacco/an-ever-tightening-stranglehold-tobacco-category-report-2018/563080.article.
26. Philip Morris International. Stock up now: Chesterfield with new premium features. Retail Newsagent. 15 December 2017.
27. Cronin E. Rizla launches new Natura paper and tips range: Talking Retail; 3 August 2017 [4 April 2018]. Available from: https://www.talkingretail.com/products-news/tobacco/rizla-launches-new-natura-paper-tips-range-03-08-2017/.
28. Appleby S. New tobacco law, means new opportunity for cigars sales: Retail Express; 6 June 2016 [24 April 2018]. Available from: https://www.betterretailing.com/cigar-market.
29. Dillon C. Ritmeester launches single stick Panatellas: Retail Express; 15 January 2015 [23 April 2018]. Available from: https://www.betterretailing.com/ritmeester-single-royal-dutch-panatellas.
30. Lee J. Know the value of the tobacco market to keep sales strong. Retail Express. 22 August 2017.
31. Gockelen-Kozlowski T. Tobacco firms adapt to deal with latest EUTPD laws: Retail Newsagent; 25 May 2017 [20 April 2018]. Available from: https://www.betterretailing.com/tobacco-legislation.
32. Ministry of Health (New Zealand). Tobacco standardised packaging 2017 [updated 13th June 2017, cited 16th March 2018]. Available from: https://www.health.govt.nz/our-work/preventative-health-wellness/tobacco-control/plain-packaging.
33. Scollo M, Lindorff K, Coomber K, et al. Standardised packaging and new enlarged graphic health warnings for tobacco products in Australia—legislative requirements and implementation of the Tobacco Plain Packaging Act 2011 and the Competition and Consumer (Tobacco) Information Standard. *Tobacco Control*. 2015;24(Suppl 2):ii9.
34. Tobacco Labelling Resource Centre. France 2013 [cited 2018 16th March]. Available from: http://www.tobaccolabels.ca/countries/france/.
35. Mutti S, Hammond D, Borland R, et al. Beyond light and mild: cigarette brand descriptors and perceptions of risk in the International Tobacco Control (ITC) Four Country Survey. *Addiction*. 2011;106(6):1166-75.
36. Bansal-Travers M, O'Connor RJ, Fix BV, et al. What do cigarette pack colors communicate to smokers in the U.S.? *American Journal of Preventive Medicine*. 2011;40(6):683-89.
37. Hoek J, Gendall P, Eckert C, et al. Effects of brand variants on smokers' choice behaviours and risk perceptions. *Tobacco Control*. 2016;25(2):160-5.
38. Greenland SJ. Cigarette brand variant portfolio strategy and the use of colour in a darkening market. *Tobacco Control*. 2015;24:e65-e71.

For peer review only - http://bmjopen.bmj.com/site/about/guidelines.xhtml

39. Borland R, Savvas S. The effects of variant descriptors on the potential effectiveness of plain packaging. *Tobacco Control*. 2014;23:58-63.
40. Hoek J, Gendall P. Policy options for extending standardized tobacco packaging. *Bulletin of the World Health Organization*. 2017;95(10):726.
41. Bayly M, Scollo MM, Wakefield MA. Who uses rollies? Trends in product offerings, price and use of roll-your-own tobacco in Australia. *Tobacco Control*. 2018:1-8.
42. Curti D, Shang C, Ridgeway W, et al. The use of legal, illegal and roll-your-own cigarettes to increasing tobacco excise taxes and comprehensive tobacco control policies: findings from the ITC Uruguay Survey. *Tobacco Control*. 2015;24:iii17-iii24.
43. Hoek J, Ferguson S, Court E, et al. Qualitative exploration of young adult RYO smokers' practices. *Tobacco Control*. 2017;26(5):563-8.
44. Doward J. How tobacco firms flout UK law on plain packaging: The Observer; 9 April 2017 [12 November 2018]. Available from: https://www.theguardian.com/society/2017/apr/09/tobacco-companies-flout-law-plain-packaging.
45. Nixon P. Re: Price marking on packs of Work. Letter from Peter Nixon, Philip Morris International to Deborah Arnott, Action for Smoking and Health. [Personal Communication]. 15 May 2017.
46. Leigh Day. Re: Price marking on tobacco packaging of Work. Legal opinion of Leigh Day to Action for Smoking and Health. [Personal Communication]. 3 March 2017.
47. Critchlow N, Stead M, Moodie C, et al. Introduction of standardized tobacco packaging during a 12-month tranistion period: findings from small retailers in the United Kingdom. *Nicotine & Tobacco Research*. 2018.

Figure captions

Figure 1. Timeline of data collection by data source

Figure 2. Proportion of (a) FM sticks and (b) RYO* (by weight) sold in standardised packaging over time

Figure 3. The evolution of Marlboro Gold packaging from a straight edge pack to a branded bevelled edge pack with a new internal packet with pro-seal sealing mechanism, to standardised packaging maintaining these innovations

Figure 4. Branded outers post May 2016 (full-implementation)

For peer review only - http://bmjopen.bmj.com/site/about/guidelines.xhtml

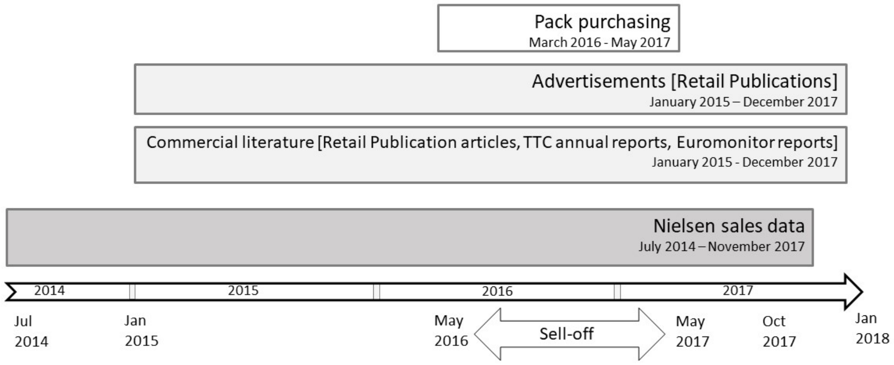

Figure 1. Timeline of data collection by data source

338x190mm (96 x 96 DPI)

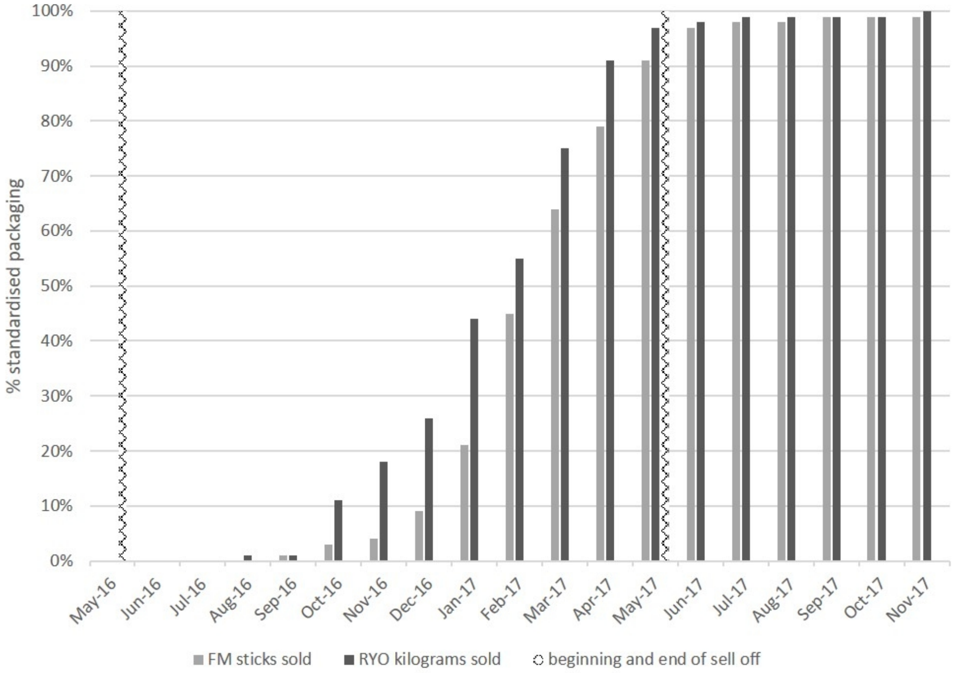

Figure 2. Proportion of (a) FM sticks and (b) RYO* (by weight) sold in standardised packaging over time

85x60mm (300 x 300 DPI)

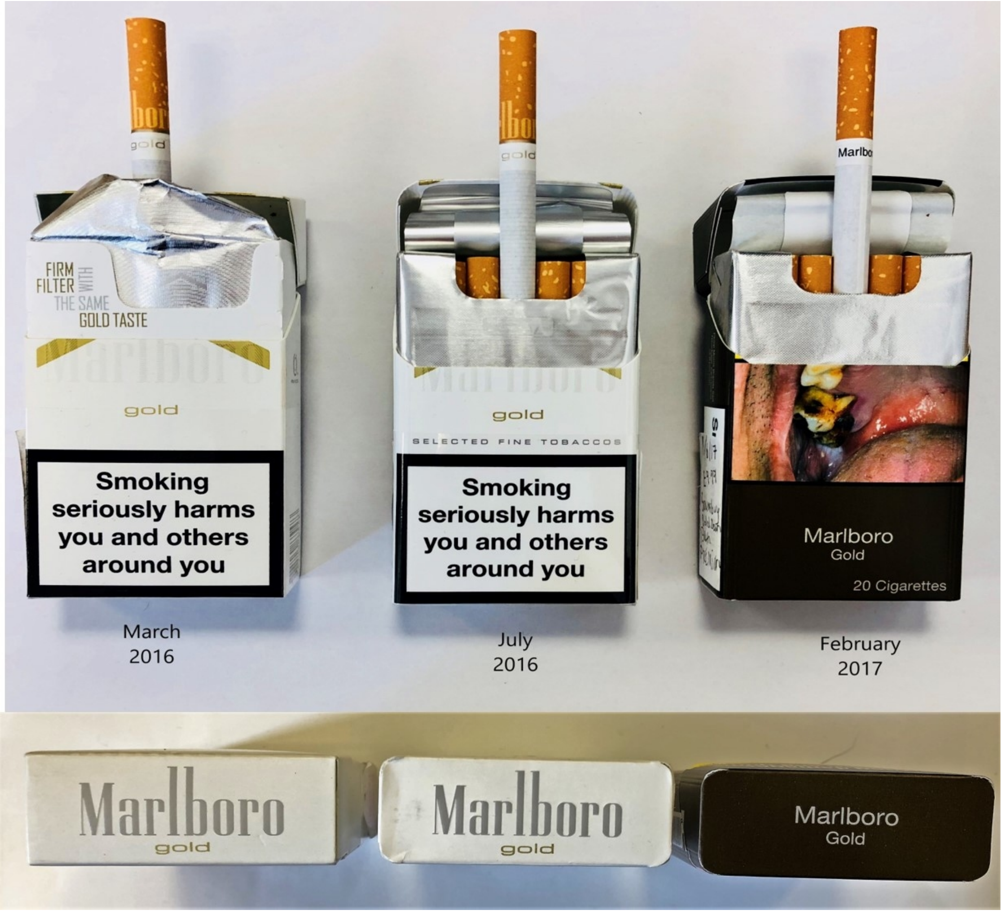

Figure 3. The evolution of Marlboro Gold packaging from a straight edge pack to a branded bevelled edge pack with a new internal packet with pro-seal sealing mechanism, to standardised packaging maintaining these innovations

103x94mm (300 x 300 DPI)

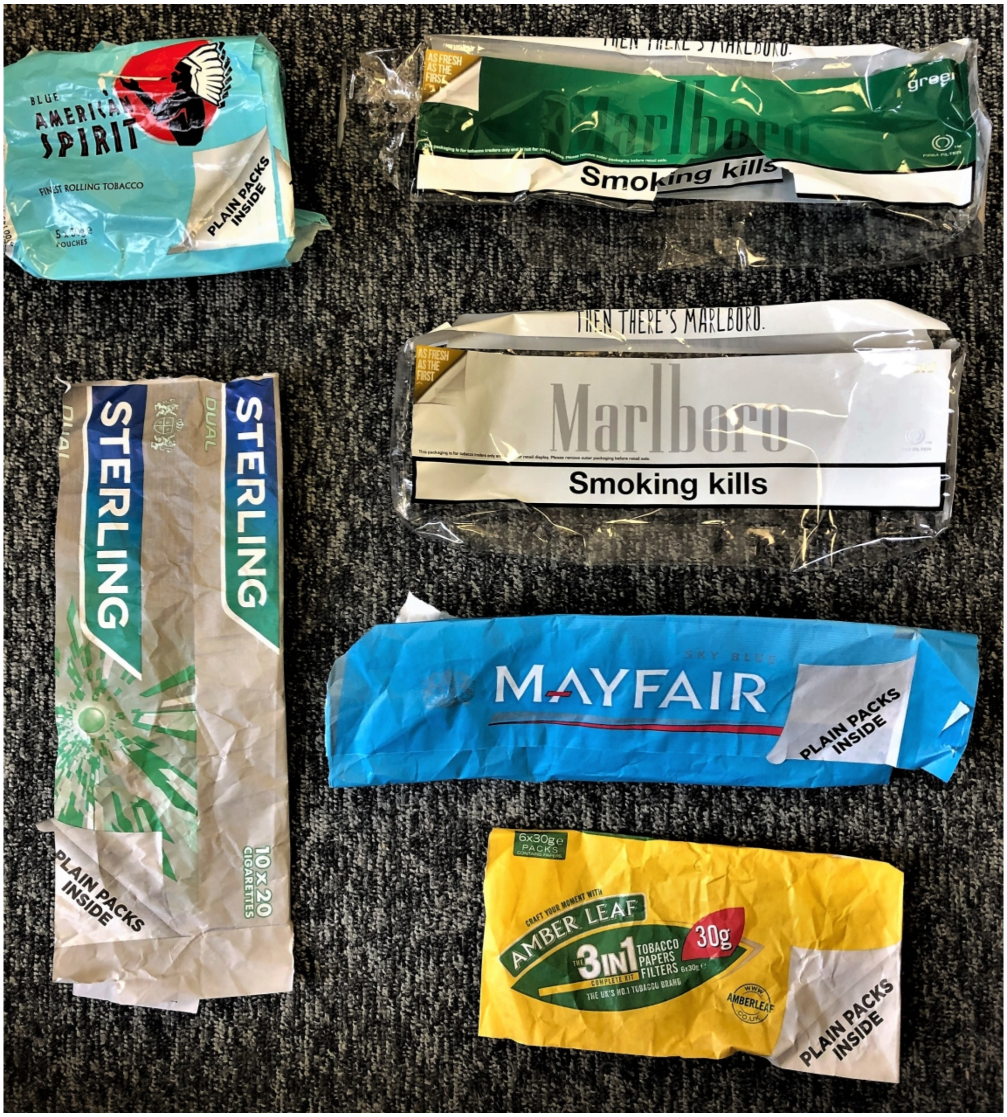

Figure 4. Branded outers post May 2016 (full-implementation)

256x283mm (300 x 300 DPI)

Supplementary file material for "Assessing tobacco company adaptation to standardised packaging: identifying circumventions and closing loopholes"

Table S1. Tobacco advert characteristics by price segment (n= 195) [1]

	FM & RYO				Cigars/ cigarillos	Pipe
Characteristics of advert[2]	Subvalue[3]	Value	Midprice	Premium		
<u>Type of tobacco</u>						
FM	22	78	42	21		
RYO	n/a	24	13	16		
<u>Characteristics promoted</u>						
New brand variant[4]	7	23	5	1	0	0
New packaging	4	25	17	16	0	0
Limited Edition	0	4	5	5	2	0
Filter	0	16	0	6	0	0
Price	19	68	14	4	2	1
Retailer profit	1	42	21	5	6	1

[1] Totals do not add up to 195 adverts as many adverts promoted more than one type of tobacco, more than one price segment and more than one characteristic in the same advert.

[2] Does not include cigars/cigarillos or pipe tobacco or as we have no data on price segment for these products.

[3] FM only. This price segment does not exist for RYO.

[4] There were 38 adverts in total for a new brand variant but 2 of the adverts could not be categorised by price segment.

For peer review only

Table S2 Industry strategies to circumvent standardised packaging and exploit loopholes implied by commercial literature

Industry strategy	Tactic	Quote from the commercial literature
1. Keep branded packaging on the market throughout the sell-off period	1.1 Over-producing branded packs before the sell-off	"BAT has had to produce higher stocks prior to the 20 May 2016 manufacturing deadline in order to avoid out of stocks [branded]…The TPD explicitly provides for transitional provisions to allow product manufactured before 20 May 2016 to be sold until 20 May 2017." Ron Ridderbeek, head of legal and external affairs at British American Tobacco [*Retail Newsagent*, 10 May 2016]
	1.2 Introducing smaller pack sizes just before the sell-off	"Tinkering with pack sizes is a proven wheeze for off setting the impact of tobacco's growing tax burden on price per pack. JTI has managed to hit the £6.50 price point by launching 17-packs of Sovereign Blue, Sky Blue, Green and Dual. And it's just in time; from May, pack sizes will be limited to a minimum of 20 cigs." Japan Tobacco International [*The Grocer,* 11 February 2016]<br><br>"Launching next month as the European Union Tobacco Productive Directive comes into force, [Pall Mall's] redesign - available in 10-pack and 18-pack King Size and Super King 18s - will include the new Taste Plus Filter … price-marked packs are set to be available at the current [price]." [*The Grocer,* 18 April 2016]
	1.3 Providing financial incentives to retailers to sell a particular company's brands	"Retailers have reported a "massive" increase in cigarette sales as tobacco manufacturers cut prices and offer cash incentives to bolster brands at a time when new plain packaging and smaller pack regulations are set to shake up the industry. … An independent retailer… said: "I have seen a massive increase in sales. We were also given PoS including shelf wobblers to encourage as many customers as possible to buy the brand. I think this is a really smart way of driving sales and is something that retailers will respond to." Meanwhile, Imperial Tobacco will knock 10p off the price of four of its biggest tobacco brands - Lambert & Butler, JPS, Players and Gold Leaf - from 1 June, while maintaining cash margins... [A retailer who] has been trialling the new Imperial prices at his store… said: "We sold up to one and a half extra outers a week because of the lower price." [*Retail Newsagent,* 26 May 2016]<br><br>"A value brands price war on cigarettes is hurting retailers' margins and could lead to stores being "lumbered" with a heap of unwanted, unsaleable stock [with legislation changes;] tobacco suppliers have been dropping the prices of cigarette packs at the value end of the market… some manufacturers… selling value cigarettes at a loss to gain market share… Last runs of branded packs are price-marked lowly and [a retailer] told Retail Express that the actions of suppliers will have grave consequences for unsuspecting retailers.<br><br>"Situations will arise where retailers are taken in by the low, price-marked RRPs and buy in bulk, thinking that consumers will jump at the chance to catch the last batches of cheap packs…Come May 20, they'll find themselves lumbered with packs of cigarettes they can no longer sell, as supply will outstrip demand…It's a

For peer review only - http://bmjopen.bmj.com/site/about/guidelines.xhtml

		unique situation, but the suppliers have stock they need to get rid of. They're cutting the prices down and loss-leading on them [but]Independent retailers certainly can't afford to loss-lead. "…[Another retailer] said: "… Companies downtrading to push their own agendas does not help us at all. I've even heard that certain wholesalers were offered better percentages to buy in these packs, which would obviously be recouped at the other end."" [*Better Retailing,* 17 February 2017]<br><br>""The tobacco suppliers will themselves take back any branded packs after the May cut-off," [wholesaler manager] told RN. "They have teams of people calling on retailers and they are notifying them as to the process. They are saying if they have any branded stock left to call their local rep and they will come in and replace it." [*Retail Newsagent*, 2 March 2017]
2. Maintain brand differentiation through systematic name changes		"We recognise that consumers use brand and colour when asking for their pack of cigarettes." Therefore, generally, those products traditionally presented as 'full flavour' would have 'real' or 'original' added to their names and be denoted predominantly by the colour 'red'. All brand variants with the flavour descriptor 'smooth' would change to 'bright' and have 'blue' added, 'menthol' would become 'green' and 'capsule' would become 'crushball'." [*Retail Newsagent*, 28 October 2016]
3. Focusing on lower price segments	Introducing premium features into cheaper price segments	"Living in a post-EUTPD II world… [a retailer reported] "Customers have become more price-sensitive since legislation banned the sale of 10- packs and they are more concerned about how much they can get for their money these days"…To help retailers tap into the trend for budget tobacco, JTI has relaunched Scottish brand Kensitas Club King Size and dropped its RRP " Yau A. The latest developments from the biggest tobacco brands. [*Retail Newsagent*, 5 April 2018]
4. Promote products by innovating exemptions to the legislation	4.1 Bevelled edges and resealable packs	"In order to re-engage disengaged cash-strapped smokers migrating to lower-priced brands and categories, manufacturers have been "adding value" to their brands through innovation, both on the pack and via the product itself… PMI concentrated on areas such as pack size (extra-large pack sizes), pack type (resealable), cigarette length (compact), microslims, filter type (with a focus on recessed filters) and flavour capsules (including the world's first recessed filter capsule and the world's first double capsule – menthol and peppermint). Pack innovation: Tactile finishes, including raised surfaces such as embossing and bevelled edges… (39)[1] [*Euromonitor*, July 2016]
	4.2 Menthol	"JPS Green Edge will replace it and feature a menthol inner liner within the packaging, which the company claims will be "more effective at transferring flavour to the cigarettes than traditional menthol tips"". Imperial Tobacco [*Talking Retail*, 24 November 2017]

[1] This Euromonitor report was published two months into the sell-off period. We did find evidence of embossing and raised surfaces on packets prior to the full implementation of the legislation but these changes were lost when standardised packaging came into effect. During the sell-off, filter innovations, capsule innovations, bevelled edges and a pack resealing mechanism on one brand were observed – these changes were able to carry through when packs became standardised. In this study we did not observe extra length cigarettes, a greater volume of cigarettes or microslims.

For peer review only - http://bmjopen.bmj.com/site/about/guidelines.xhtml

	4.3 Filters (another way to create the perception that some products are healthier than others?)	Marlboro Touch, previously known as Marlboro Gold Touch, now has a soft touch pack and firm filter keeping the consistency with the rest of the Marlboro range. The firm filter allows adult smokers a cleaner way to stub out their cigarette, as well as adding an overall feeling of quality, without having an impact upon taste." [*Talking Retail,* 31 July 2015]<br><br>"Launching …as the European Union Tobacco Productive Directive comes into force, [Pall Mall's] redesign… will include the new Taste Plus Filter that aims to create a firmer in-hand feel and improve filtration" [*The Grocer,* 18 April 2016]<br><br>"…JPS Triple Flow… "a revolution in cigarette technology." Triple Flow uses "unique" "easy draw" channels, with a new, "high quality smooth" tobacco blend enhanced by a mineral filter that provides "a fuller smoking experience". Each cigarette is also wrapped in a special paper, designed to actively reduce the amount of smoke emitted - which may result in less smell, says Imperial. Brand portfolio manager Emma Stew said: "Smooth blends now account for almost a quarter [ITUK estimates September 2015] of factory made cigarette (FMC) sales, and Triple Flow has been designed to offer the ultimate smooth smoking experience. 'With its next generation features and distinctive packaging, there is nothing like it in the market'" [*Wholesale News* November 2015]
	4.4 RYO papers and filter tips	Imperial Tobacco brand manager Jamie Burns-Smith said: "Natura taps into an increasing consumer desire for responsibly sourced products. Crafted from 100% hemp, Natura papers and tips are the perfect sustainable way to enjoy an authentic tobacco taste. Imperial Tobacco has also launched Rizla Menthol Tips, as the company claims "menthol tips are the fastest-growing segment of the tips market". [*Talking Retail,* 3 August 2017]
	4.5 Cigars	"Break, as they are cigarillos, are exempt. We can do packs of less than 20, and we can do flavours… Cigars won't be a standard colour pack, and will still have product name branding… you can sell individually and in 10 packs" Jens Christiansen, Scandinavian Tobacco Group's head of marketing & public affairs [*Retail Express*, 6 June 2016]<br><br>"Cigars benefit from the fact they can be sold in 10-packs, while also being exempt from certain branding restrictions. Scandinavian Tobacco Group (STG) is using this to its advantage and its marketing this year… the packaging stands out when compared to traditional cigarettes… and the investment made by companies such as STG to raise awareness and education in the convenience trade really helps... Ritmeester's says miniature cigars have traditionally been products that are better known among tobacco enthusiasts. As a result, it is offering samples of its Royal Dutch range to retailers to increase awareness…

For peer review only - http://bmjopen.bmj.com/site/about/guidelines.xhtml

		"I find the curry socials Ritmeester holds with retailers and the trade press very useful. It's a hands-on, but informal way of learning about the latest developments in the cigar market. There isn't as much awareness about cigars as cigarettes. These opportunities give us a chance to learn about how we can upsell to smokers who want value." [Retailer]" [*Retail Newsagent*, 5 April 2018]<br><br>"If a smoker only has £5 on them, they cannot buy from your gantry, but if you present them with cigars they can." Ritmeester [*Retail Express*, 17 September 2017]<br><br>"[Retailer] says he struggles with selling Mini Moods. [Ritmeester's] Lyles explains: "It might be because you're aiming them at cigar smokers, but it's primarily aimed at cigarette smokers. Because of the double filter, it makes for a smoother smoke." … Cigars are now the cheapest product in a gantry. Lyles says: "If a smoker only has £5 on them, they cannot buy from your gantry, but if you present them with cigars they can." [*Retail Express*, 17 September 2017]<br><br>"'The margins we make on miniature cigars are 30% when sold at RRP' [Retailer]" [*Retail Newsagent*, 5 April 2018]
	4.6 Pipe tobacco sold as RYO	"The sell-through period for branded RYO and cigarettes ended last week, with products now sold in minimum formats and carrying larger health warnings as part of the new tobacco legislation. Yet, UK tobacco manufacturer Gawith Hoggarth is promoting its Kendal brand as a product which is cut to pipe tobacco regulations but can also be used as hand-rolling tobacco. "We've cut the tobacco to the thinnest width we are allowed to which means we can sell it in branded packaging and in 12.5g pouches," said the company's area sales manager Martin Bobbette. The company hopes retailers will recommend the product to smokers who ask for "whatever is cheapest"" [*Retail Newsagent*, 25 May 2017]

For peer review only - http://bmjopen.bmj.com/site/about/guidelines.xhtml

Table S3 Compliance with standardised packaging over time[1]

	% standardised packs	change from previous month	total sticks sold	non-compliant sticks sold
Jul-16	0%		2,574,340,308	2,574,204,015
Aug-16	0%	0%	2,620,104,622	2,610,727,340
Sep-16	1%	1%	2,424,692,602	2,397,886,927
Oct-16	3%	2%	2,491,577,487	2,423,864,794
Nov-16	4%	1%	2,424,660,803	2,323,057,266
Dec-16	9%	4%	2,593,371,625	2,370,713,888
Jan-17	21%	12%	2,316,632,301	1,829,871,306
Feb-17	45%	24%	2,184,811,712	1,204,205,686
Mar-17	64%	20%	2,402,514,817	852,983,422
Apr-17	79%	14%	2,386,041,031	506,014,392
May-17	91%	12%	2,475,338,965	228,361,777
Jun-17	97%	6%	2,359,196,539	81,803,934
Jul-17	98%	1%	2,461,000,857	53,453,290
Aug-17	98%	0%	2,412,267,420	43,336,437
Sep-17	99%	0%	2,267,864,526	29,804,540
Oct-17	99%	0%	2,303,639,526	27,803,694
Nov-17	99%	0%	2,231,013,199	25,718,138
RYO			total thousands of kg sold	non-compliant* (thousands of kg sold)
Aug-16	1%		552287	546963
Sep-16	1%	0%	521917	516721
Oct-16	11%	10%	528560	469493
Nov-16	18%	7%	518188	424040
Dec-16	26%	8%	541359	400109
Jan-17	44%	18%	516174	288710
Feb-17	55%	11%	490464	218940
Mar-17	75%	20%	539695	132601
Apr-17	91%	16%	534343	45509
May-17	97%	5%	557734	19294
Jun-17	98%	2%	539118	9037
Jul-17	99%	1%	559731	5908
Aug-17	99%	0%	560994	4609
Sep-17	99%	0%	536870	2984
Oct-17	99%	0%	542052	2802
Nov-17	100%	0%	530219	2511

*RYO estimated to be sold in standardised packaging if sold in 30g or larger not pricemarked pack and (a) 30g pack of same variant on the market or (b) if 30g pack never available, pack on the market at end of analysis period)

For peer review only

For peer review only - http://bmjopen.bmj.com/site/about/guidelines.xhtml

Table S4 Menthol and capsule innovation

	Present at start		New Variant		Total	
	N	%	N	%	N	%
Menthol*						
FM premium	9	28%	2	20%	11	26.2
FM midprice	8	25%	0	0%	8	19.0
FM value	8	25%	4	40%	12	28.6
FM subvalue	3	9%	3	30%	6	14.3
no price segment	4	13%	1	10%	5	11.9
	32	76.2%	10	23.8%	42	100.0
Capsule**						
FM premium	2	15%	0	0%	2	10.5
FM midprice	2	15%	0	0%	2	10.5
FM value	9	69%	3	50%	12	63.2
FM subvalue	0	0%	3	50%	3	15.8
Total	13	68.4%	6	31.6%	19	100.0

*Menthol: variant name includes 'menthol' or 'green'
**Capsule: variant name includes 'capsule', 'crushball', 'dual', 'duo', 'demand', 'click on' or 'burst'

Table S5 Euromonitor estimates of the growth of cigars and cigariillos

	value constant £ mln	value current £ mln	volume million units	
2015	291.8	289.9	390.4	
2016	282.5	282.5	382.8	
2017	324.5	332.6	451.6	estimate
2018	347.8	366.3	493.8	estimate

Source: © Euromonitor International

For peer review only
